# MEF2C opposes Notch in lymphoid lineage decision and drives leukemia in the thymus

**DOI:** 10.1172/jci.insight.150363

**Published:** 2022-07-08

**Authors:** Kirsten Canté-Barrett, Mariska T. Meijer, Valentina Cordo’, Rico Hagelaar, Wentao Yang, Jiyang Yu, Willem K. Smits, Marloes E. Nulle, Joris P. Jansen, Rob Pieters, Jun J. Yang, Jody J. Haigh, Steven Goossens, Jules P.P. Meijerink

**Affiliations:** 1 Princess Máxima Center for Pediatric Oncology, Utrecht, The Netherlands.; 2 Department of Pharmaceutical Sciences, St. Jude Children’s Research Hospital, Memphis, Tennessee, USA.; 3 Research Institute in Oncology and Hematology, CancerCare Manitoba, Winnipeg, Manitoba, Canada.; 4 Department of Pharmacology and Therapeutics, Rady Faculty of Health Sciences, University of Manitoba, Winnipeg, Manitoba, Canada.; 5 Biomolecular Medicine, Ghent University, Ghent, Belgium.

**Keywords:** Hematology, Oncology, Cancer, Leukemias, T cell development

## Abstract

Rearrangements that drive ectopic MEF2C expression have recurrently been found in patients with human early thymocyte progenitor acute lymphoblastic leukemia (ETP-ALL). Here, we show high levels of MEF2C expression in patients with ETP-ALL. Using both in vivo and in vitro models of ETP-ALL, we demonstrate that elevated MEF2C expression blocks NOTCH-induced T cell differentiation while promoting a B-lineage program. MEF2C activates a B cell transcriptional program in addition to RUNX1, GATA3, and LMO2; upregulates the IL-7R; and boosts cell survival by upregulation of BCL2. MEF2C and the Notch pathway, therefore, demarcate opposite regulators of B- or T-lineage choices, respectively. Enforced MEF2C expression in mouse or human progenitor cells effectively blocks early T cell differentiation and promotes the development of biphenotypic lymphoid tumors that coexpress CD3 and CD19, resembling human mixed phenotype acute leukemia. Salt-inducible kinase (SIK) inhibitors impair MEF2C activity and alleviate the T cell developmental block. Importantly, this sensitizes cells to prednisolone treatment. Therefore, SIK-inhibiting compounds such as dasatinib are potentially valuable additions to standard chemotherapy for human ETP-ALL.

## Graphical abstract

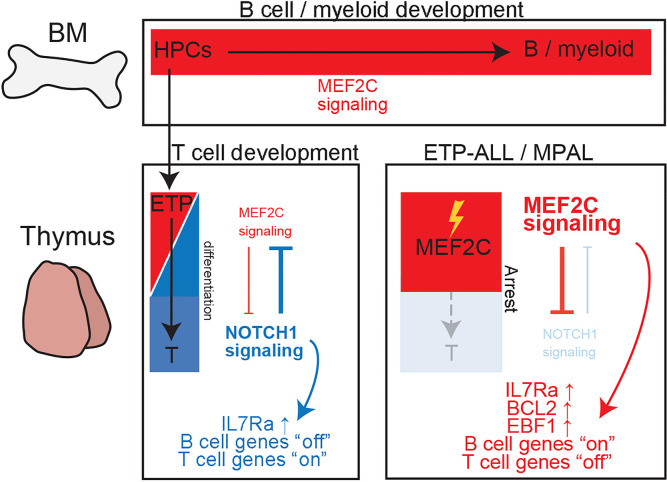

## Introduction

T cell acute lymphoblastic leukemia (T-ALL) is a cancer of early thymocytes and is characterized by recurrent chromosomal rearrangements that activate oncogenic transcription factors such as *TAL1/2*, *LMO1/2/3*, *NKX2.1/2.2*, *TLX1*, *TLX3*, or *HOXA*. These oncogenic transcription factors facilitate developmental arrest at specific thymocyte maturation stages (1–5). Extensive sequencing efforts have resulted in the identification of additional putative driver mutations, gene fusions, or deletions, including *SPI1* (encoding PU.1), *MEF2C*, *CCND3*, *MYB*, and *MYCN* (6–8). Whole-genome gene expression analysis led to the identification of 4–5 biologically distinct subtypes among patients with T-ALL (i.e., the TALLMO, Proliferative, TLX, and immature subtypes) that each harbor a specific gene expression signature and are almost exclusively characterized by unique oncogenic rearrangements (2, 3, 5). More recent whole exome and RNA-Seq strategies characterized T-ALL subgroups based on the presence of specific oncogenic rearrangements (6, 9). Aside from such driving oncogenes, additional mutations in many other genes that affect cytokine and receptor signaling, transcriptional regulation, cell cycle, apoptosis, chromatin modifications, or transporters have been recurrently identified in T-ALL (6, 9–16). Patients with early thymocyte progenitor–ALL (ETP-ALL) reflect the most immature T-ALL entity (1) and contain recurrent lesions that activate *MEF2C* (5, 9) or *HOXA* genes (6, 9) or that generate *PU.1* fusions (7). ETP-ALL has been associated with poor clinical outcome in various pediatric and adult patient studies (1, 17–20), but not in all (21–23), and adult ETP-ALL patients with HOXA-activating events have been linked with poor outcome (24). ETP-ALL is characterized by an active hematopoietic stem cell renewal program and high expression of *LYL1*, *LMO2*, *MEF2C*, *HHEX*, and *BCL2* (1, 5, 25, 26).

Myocyte Enhancing Factor 2 (MEF2) proteins MEF2A–D are part of the MADS box (MCM1-Agamous-Deficiens-Serum response factor) family of transcription factors that contain MEF and MADS domains. MEF2C is a regulator of muscle cell development (27) and has been implicated in the development of various tissues, including hematopoietic lineages (28, 29). Murine KO studies have elucidated that MEF2C directs the lineage choice from common myeloid to common lymphoid progenitors, initiates pre–B cell development, and mediates B cell receptor–driven survival, as well as proliferation signals (30–34). MEF2C is not expressed in normal T cells (29, 35). MEF2C regulates target genes upon binding to MEF binding sites in regulatory enhancers. MADS and MEF domains are essential for DNA binding, dimerization, and binding to coactivators EP300/CBP (36). Class II histone deacetylases HDAC4/5 are important negative regulators of MEF2C that also bind to the MADS-MEF interface (37) and drive local histone deacetylation and chromatin compaction. HDAC4 and CDK5 negatively regulate MEF2C by sumoylation (38), whereas phosphorylation of HDAC4/5 by salt-inducible kinases (SIK) (39, 40) enhance MEF2C’s function. MEF2C is also directly positively regulated upon phosphorylation by various kinases, including microtubule affinity regulating kinase 2 and 3 (MARK2/3), calcium /calmodulin-dependent protein kinase 1a (CaMK1a), or mitogen-activated protein kinase p38 (41–48).

Since we identified MEF2C as putative oncogene for human ETP-ALL (5), we investigated the significance of ectopic MEF2C expression in early T cells in relation to T cell differentiation and pathogenesis. For this, we generated and analyzed conditional knockin mice harboring a *MEF2C-eGFP* transgene that is activated upon *Lck*-Cre activation in early thymocytes and T cells. Furthermore, inducible MEF2C expression or KO ETP-ALL models were developed from the LOUCY cell line to further study the effect of MEF2C on gene regulation, differentiation, growth, and survival in-depth and to identify clinically relevant inhibitors that can impair its function.

## Results

### MEF2C is highly expressed in ETP/immature patients with T-ALL.

**Figure 1:**
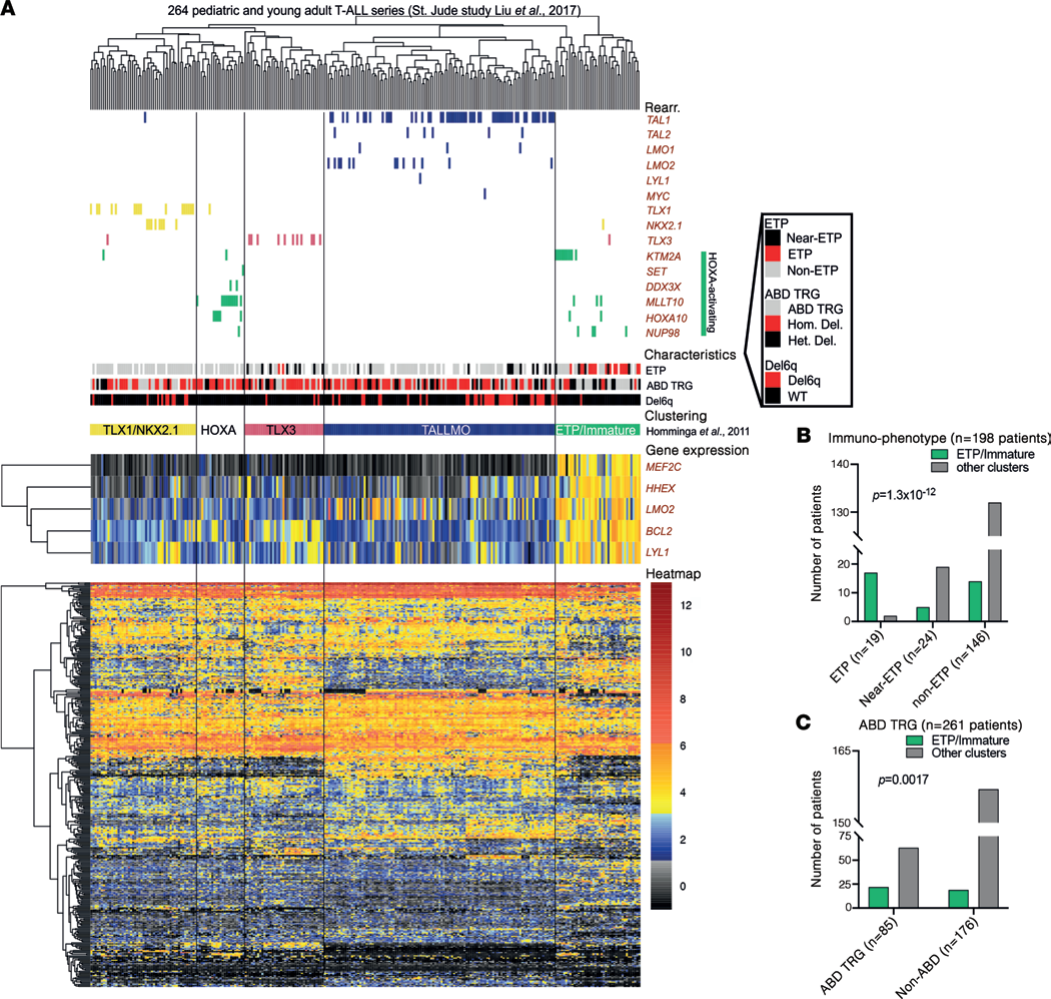
ETP/immature subtype pediatric and young adult patients with T-ALL highly express *MEF2C* and other stem cell factors *HHEX*, *LMO2*, *BCL2*, and *LYL1*. (**A**) Cluster analysis of 264 pediatric and young adult patients with T-ALL from the St. Jude study by Liu et al. (6) based on 371 available genes out of the 416-gene set (435 probe sets) as originally defined in the unsupervised cluster analysis of 117 pediatric patients with T-ALL by Homminga et al. (5). Specific clusters have been indicated as TLX1/NKX2.1 (formerly denoted as proliferative subtype), HOX, TLX3 (both formerly included in the TLX cluster), TALLMO, and ETP/immature. Specific oncogenic rearrangements are indicated for driving oncogenes, and patient characteristics include immunophenotypic ETP, near ETP, and non-ETP patients. The absence of biallelic TRG deletions (ABD) are indicated as reported before (6). (**B** and **C**) Distribution of ETP, near ETP, and non-ETP cases or ABD and non-ABD patients with T-ALL over the ETP/immature cluster versus other T-ALL clusters.

Using gene expression profiling of pediatric patients with T-ALL, we previously identified 4 biological T-ALL subtypes, among which was an immature T-ALL subtype with *MEF2C*-activating rearrangements in some of the patients (5), in extension of previous observations by others (2, 3). This immature entity was highly reminiscent of immunophenotypically defined patients with ETP-ALL (1, 23), and about half of the cases in the immature subtype had MEF2C-activating rearrangements (5). To validate these findings and high expression of *MEF2C* in ETP/immature patients, we used our original gene set based on 416 genes (435 probe sets) to predict specific T-ALL subtypes in an extended cohort of pediatric and young adult patients with T-ALL (*n* = 264) that has been well annotated genetically using whole-exome and whole RNA-Seq (6). Based on the expression of 371 overlapping genes as expressed in the RNA-Seq data set (Supplemental Table 1; supplemental material available online with this article; https://doi.org/10.1172/jci.insight.150363DS1), patients with T-ALL cluster into separate biologically clusters that are almost indistinguishable from those as originally identified (5) (Figure 1A); most *TLX1*- or *NKX2.1*-rearranged patients fall into a single cluster (previously denoted as proliferative), and some patients with *HOXA*-activating events cluster into a separate cluster, while the third cluster comprises all except 2 *TLX3*-rearranged patients. The TALLMO cluster comprises all patients with *TAL1/2*, *LMO1/2*, *LYL1*, and *MYC* translocations except 1. The ETP/immature cluster as identified comprises most *KTM2A*-rearranged patients, along with patients having other *HOXA*-activating events as observed before (5, 24). It also comprises various patients for which underlying oncogenic events have not been resolved. This cluster strongly overlaps with patients who display an ETP-ALL (*P* = 1.3 × 10^–12^) but not a near–ETP-ALL immunophenotype and who frequently lack *TRG* recombination events (*P* = 0.0017; Figure 1, B and C) (6). This cluster also strongly and significantly associates with expression of a stem cell signature that includes *MEF2C*, *BCL2*, *HHEX*, *LMO2*, and *LYL1* (Supplemental Figure 1, A–E). MEF2C expression levels correlate with *BCL2* and *LMO2* expression levels in patients with ETP-ALL but not in other patients with T-ALL (Supplemental Figure 1F), and it is validated for *LMO2* in the original data set as published by Homminga et al. (5) (Supplemental Figure 1G). Recurrent *MEF2C*-driving events as identified by us before (5) may explain, in part, the high expression levels of *MEF2C* as putative oncogene for patients with ETP-ALL.

### MEF2C antagonizes Notch signaling in early progenitor T cells.

**Figure 2:**
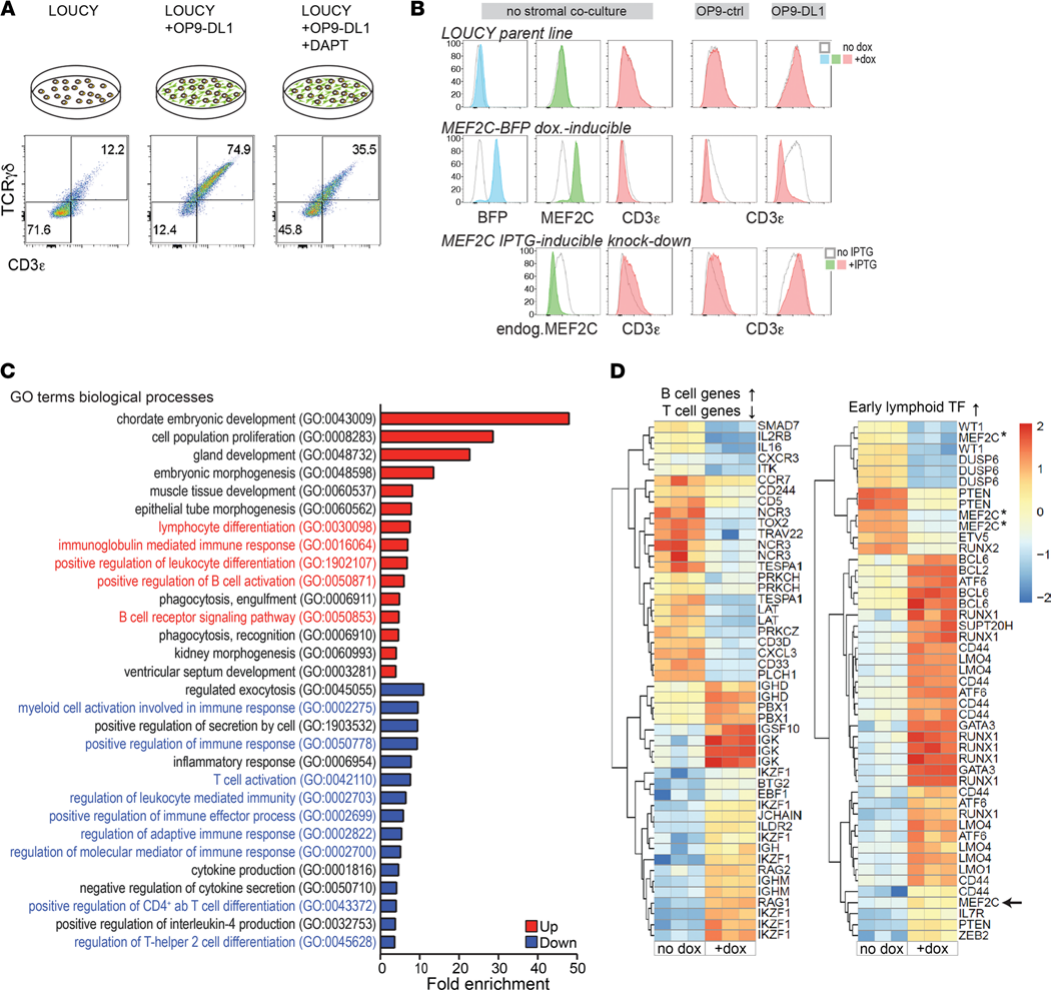
MEF2C antagonizes Notch signaling in early progenitor T cells and induces B cell gene expression. (**A**) TCRγδ/CD3ε surface expression on LOUCY cells that were cultured in media or cocultured for 3 days on OP9-DL1 stromal cells, in the absence or presence of 10 μM of γ-secretase inhibitor DAPT visualized by flow cytometry (*n* = 3). (**B**) Transduced LOUCY cells containing a dox-inducible MEF2C-BFP overexpression construct (middle panels) or an IPTG-inducible MEF2C shRNA knockdown construct (bottom panels) were generated and compared with parental LOUCY cells (top panels). Blue fluorescence (BFP, blue-filled histograms), intracellular MEF2C levels (green-filled histograms), and surface CD3ε levels (red-filled histograms) were analyzed using flow cytometry in the absence (open histograms) or presence of dox or IPTG, respectively (filled histograms), as indicated. “Endog. MEF2C” refers to endogenous MEF2C expression levels in the MEF2C shRNA knockdown line. CD3ε expression after coculture on OP9 control or OP9-DL1 stromal cells (right columns). (**C**) Significantly enriched up- (red) or downregulated (blue) GO terms in MEF2C-induced LOUCY cells for 24 hours compared with noninduced LOUCY cells. (**D**) Heatmaps of significantly up- or downregulated probe sets (log_2_ fold change >0.6, *P* < 0.05, FDR < 0.1) in triplicate gene expression analysis (Affymetrix GeneChip Human Genome U133 Plus 2.0) per condition for LOUCY_MEF2C-BFP cells with/without MEF2C induction for 24 hours (± dox). Downregulated or upregulated probe sets following MEF2C-induction are shown in blue or red, respectively. Three of the 4 *MEF2C* probe sets, designated with asterisks, lie outside the cloned MEF2C cDNA sequence and thus represent endogenous *MEF2C* levels, whereas the other *MEF2C* probe set (indicated by the arrow) also covers the cloned cDNA construct.

To functionally explore the significance of MEF2C in patients with ETP-ALL, we used the LOUCY line, which has an immature T-ALL phenotype that expresses *MEF2C* due to a chromosomal rearrangement near the *MEF2C* locus at chromosomal band 5q14.3 and that lacks *NOTCH1* or *FBXW7* mutations (13, 15). Whereas most LOUCY cells have an immature immunophenotype, these cells retain a low potential to differentiate into the γδ-lineage, and approximately 12% of LOUCY cells adopt a TCRγδ^+^/CD3ε^+^ phenotype in tissue culture (Figure 2A). This is a Notch-dependent process, as Notch activation by coculturing LOUCY cells on Notch Delta-like 1 ligand^+^ (DL1 ligand^+^) OP9 stromal cells (OP9-DL1) induces a TCRγδ^+^/CD3ε^+^ phenotype in nearly 75% of LOUCY cells. This is largely blocked by the Notch inhibitor DAPT (Figure 2A). To functionally explore the function of MEF2C, we generated bulk LOUCY lines that contain a doxycycline-inducible (dox-inducible) *MEF2C* expression construct or an isopropyl β-d-1-thiogalactopyranoside–inducible (IPTG-inducible) shRNA *MEF2C*–knockdown construct. Knockdown of *MEF2C* in LOUCY strongly increases the percentage of TCRγδ^+^/CD3ε^+^ LOUCY cells in culture, and this is further potentiated by coculturing on OP9-DL1 stromal cells (Figure 2B). In a reciprocal setting, induction of MEF2C expression blocks differentiation, even in the presence of active Notch signaling (OP9-DL1). Thus, MEF2C induces an early T cell differentiation arrest that overrides Notch signaling, which suggests that MEF2C and NOTCH1 represent antagonistic or competitive signaling pathways in ETP-ALL. Although Notch signaling can antagonize MEF2C-driven myogenesis by competitive recruitment of MAML1 (49), we found no evidence that MEF2C binds to MAML1 directly or to cleaved intracellular NOTCH1 (ICN1; Supplemental Figure 2A). MEF2C also does not affect NOTCH1 receptor density on the cell surface (Supplemental Figure 2B) or alter ICN levels in cytoplasmic or nuclear compartments (Supplemental Figure 2C and data not shown). Therefore, MEF2C overrides NOTCH1-induced differentiation of early ETP cells indirectly.

### MEF2C regulates early lymphoid transcription factors and B cell genes while inhibiting T cell genes.

MEF2C propagates a B cell development program at the expense of T cell development in early developing T cells, as illustrated by gene expression profiling of our LOUCY models. Upon induction of MEF2C, there was a strong upregulation of genes involved in B cell development, including immunoglobulin genes *IKZF1* and *RAG*, in addition to activation of embryonic and muscle development programs (Figure 2C). T cell genes including *LAT*, *ITK*, *TESPA1*, *IL2RB*, *CD3D*, and *CD5* are strongly repressed despite an ETP-ALL cellular context (Figure 2D, Supplemental Figure 3, and Supplemental Table 2). MEF2C induces expression of important hematopoietic and/or early B cell transcriptional regulators including *EBF1*, *RUNX1*, *GATA3*, *LMO1*, *LMO4*, and *BCL6*, but not *PAX5*. Moreover, MEF2C upregulates genes that are highly expressed in ETP and ETP-ALL, including *IL7Ra* and *BCL2* (Figure 2D and Supplemental Figure 3). Therefore, these genes may represent direct MEF2C target genes. MEF2C induces expression of *IL7Ra*, *BCL2*, and *GATA3* but represses expression of *CD5* and endogenous *MEF2C* (Supplemental Figure 4A). In line with repression of the extracellular TCRγδ/CD3ε receptor complex following induction of MEF2C, the *CD3e* mRNA level is also reduced (Supplemental Figure 4A).

IL-7 signaling is important for early lymphoid development, and ectopic signaling contributes to ETP-ALL and T-ALL (14, 16, 50). Consistent with this crucial role in healthy and malignant T cells, *IL7Ra* has been identified as a direct Notch target gene (51). However, *IL7R* expression is also upregulated upon induction of MEF2C in LOUCY cells (Supplemental Figure 4A). This is further upregulated during OP9-DL1 cocultures (Supplemental Figure 4B), with strong IL-7R induction in both the NOTCH1-induced TCRγδ^+^/CD3ε^+^ cells that escaped MEF2C-mediated TCRγδ/CD3ε repression, as well as in MEF2C (BFP^+^) TCRγδ^–^/CD3ε^–^ cells (Supplemental Figure 4C). We therefore conclude that IL-7R signaling in ETP cells is regulated by MEF2C during early development and is continued by Notch signaling during normal T cell development in the thymus.

### MEF2C binds enhancers, which is reduced by Notch signaling.

**Figure 3:**
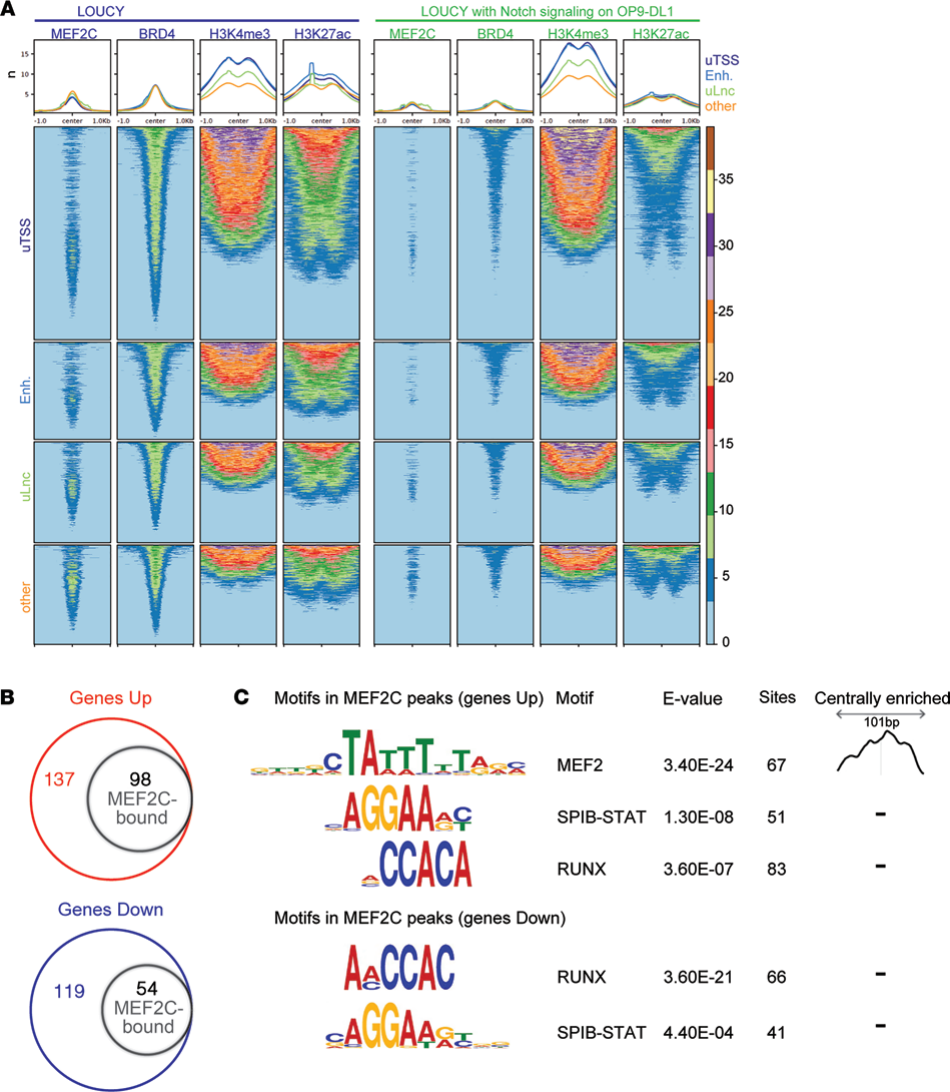
MEF2C binds to promoters and enhancers, which is reduced by Notch signaling. (**A**) Color-coded centered heatmaps indicating ChIP-Seq peak binding intensities (number of reads in a –1.0 kb to +1.0 kb region relative to the summit of the peak) for MEF2C, Bromodomain 4 (BRD4), and histone H3 with epigenetic trimethylation (H3K4me3) or acetylation marks (H3K27ac) for LOUCY parental cells (blue) or LOUCY parental cells cultured on OP9-DL1 stromal cells for 24 hours (green). Centered heatmaps for 5 kb regions upstream of transcription start site (uTSS), GeneHancer-defined enhancers (Enh), 5 kb regions upstream of long noncoding areas (defined by deepbase and lncipedia [uLnc]), or other regions (Supplemental Figure 3). (**B**) The number of upregulated genes (from collapsed probe sets; red circle) and downregulated genes (blue circle) with a log_2_ fold change > 0.6 (with *P* < 0.05 and *q* < 0.1) 24 hours after induction of MEF2C expression are displayed (see also Figure 2, C and D). The smaller black circles indicate the number of genes having MEF2C binding sites within the gene body or within 10 kb flanking regions. (**C**) Motifs as identified by MEME-ChIP found within 50 bp upstream or downstream regions relative to the MEF2C ChIP-Seq peak summits (101 bp window) are indicated including their expectancy values (*E* value), the number of sites identified, and their relative locations in these windows.

We then performed MEF2C ChIP-Seq experiments to confirm its role as a transcriptional regulator for many genes that are differentially regulated following induction of MEF2C in LOUCY cells. Endogenous MEF2C binding is observed near active promoters and enhancers (H3K27ac) in conjunction with BRD4 in LOUCY parental (Figure 3A) and noninduced LOUCY_MEF2C-BFP cells (–dox, data not shown). Induction of MEF2C (+dox) strongly increases its binding to these sites. While Notch activation by culturing LOUCY cells on OP9-DL1 stroma does not impact active H3K4me3, it lowers the binding of endogenous MEF2C and BRD4 at promoters and enhancers, and it diminishes local H3K27 acetylation (Figure 3A). These global changes in the MEF2C-regulated chromatin landscape are consistent with large changes in gene expression levels and reflect changes in cell identities such as observed between normal multipotent progenitors/ETP cells versus Notch-induced, T lineage–committed thymocyte subsets in the thymus (52, 53). We then compared the overlap between differentially expressed genes in LOUCY with and without induction of MEF2C expression with the ChIP-Seq results for genes that contain MEF2C binding sites within a 10 kb distance; roughly half of MEF2C-upregulated genes but only one-third of MEF2C-downregulated genes contain or are flanked by MEF2 binding sites (Figure 3B). MEF2 motifs are centrally enriched at these binding peaks (with a 101 bp window), along with significant enrichment for SPIB-STAT and RUNX binding sites that flank MEF2 sites in both up- and downregulated genes (Figure 3C). This may point to potential collaboration between MEF2C and RUNX1 transcriptional regulators in normal ETP and malignant ETP-ALL cells. Among upregulated genes, MEF2C-binding peaks correspond with BRD4 and H3K27ac binding peaks in enhancer areas of the important ETP genes, including *IL7R* and *BCL2* (Supplemental Figure 5). For the *IL7R* gene, the MEF2C-binding enhancer peak is located in the *CAPSL* locus and overlaps with the published NOTCH1 binding sites in CUTLL1 cells (54). Interestingly, another major NOTCH1 binding site in the *IL7R* enhancer is located just downstream of *CAPSL* (54) but is not bound by MEF2C. Future studies may show how NOTCH1 and MEF2C interact with these enhancers and regulate gene expression.

### MEF2C induces BCL2 and provides a survival advantage under limiting serum levels.

**Figure 4:**
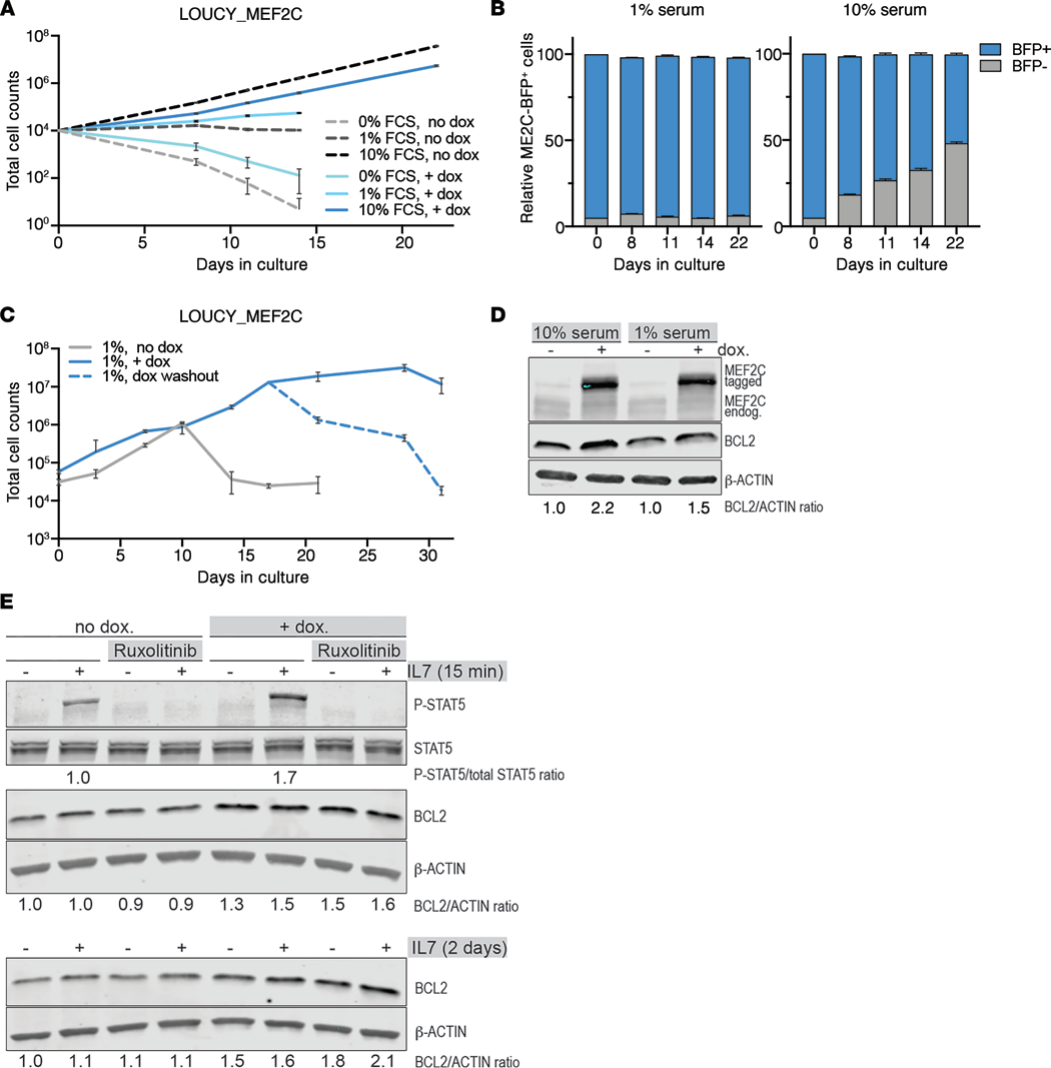
MEF2C induces BCL2 and provides a survival advantage under limiting serum levels. (**A**) LOUCY_MEF2C-BFP cells were cultured in triplicate without (dashed lines) or following induction of MEF2C (+dox, solid lines) in media containing 0%, 1%, or 10% fetal calf serum (FCS; mean values ± SD are shown). (**B**) Percentage of cells expressing the MEF2C-BFP construct in the derivative LOUCY_MEF2C-BFP bulk line. Triplicate experiments grown for 22 days in the presence of 1% (left) or 10% (right) FCS. Each column displays the fraction of MEF2C-BFP expressing cells in blue. (**C**) LOUCY_MEF2C-BFP cells were grown in medium containing 1% FCS without (–dox, gray line) or with MEF2C-BFP induction (+dox, blue line). Dox was washed out at day 17 (dashed blue line), and cells were further cultured in 1% FCS media without dox. Mean values ± SD from an experiment in triplicate are shown; shown are representative examples of 3 independent experiments performed. (**D**) Western blot for MEF2C and BCL2 in LOUCY_MEF2C-BFP cells that were grown in 1% or 10% FCS media for 3 days without (–dox) or with MEF2C induction (+dox). Relative band intensities for BCL2 normalized to β-actin are indicated, with the –dox condition ratio set at 1. (**E**) Western blot of phospho-STAT5 (Tyr694), total STAT5, and BCL2 in LOUCY_MEF2C-BFP cells that were grown in 10% FCS media without (–dox) or with MEF2C induction (+dox) for 24 hours and subsequently incubated without and with 100 ng/mL IL-7 for 15 minutes (top) or 2 days (bottom). Ruxolitinib (2 μM) was added for 1 hour before IL-7 addition and remained present throughout the experiment. β-Actin was used as a loading control. Relative band intensity ratios for phospho-STAT5 (normalized to total STAT5 level) and for BCL2 (normalized to β-actin) are shown as explained in **D**.

As MEF2C directly regulates BCL2, we then tested whether induced expression of MEF2C can rescue LOUCY cells from serum starvation. MEF2C-induced cells grow slightly slower in 10% serum medium than noninduced LOUCY-MEF2C control cells (Figure 4A) or parental LOUCY cells (not shown). However, at limiting serum conditions (1% serum), MEF2C-induced cells survive and continue proliferation, whereas noninduced LOUCY or parental controls die (Figure 4A). Knockdown of endogenous MEF2C through an IPTG-inducible shRNA construct did not enhance the sensitivity to serum-limiting conditions (not shown). In 10% serum conditions, MEF2C-induced LOUCY cells are slowly overgrown by a low percentage of cells that are refractory to MEF2C induction. This does not occur under limiting serum conditions (Figure 4B). Dox washout at day 17 for LOUCY_MEF2C-BFP cells and continued culturing under limiting serum conditions lead to rapid cell death (Figure 4C). As a potential MEF2C target gene, we found that BCL2 levels increase in MEF2C-induced LOUCY cells, both at normal (10%) and limiting (1%) serum conditions compared with noninduced controls (Figure 4D) or parental LOUCY cells (not shown). Since BCL2 is also regulated through STAT5 downstream of IL-7–induced signaling, we measured BCL2 levels in the absence and presence of IL-7 in both noninduced and MEF2C-induced LOUCY cells. Following IL-7 administration, phospho-STAT5 levels are readily detected and are higher for MEF2C-induced LOUCY cells than noninduced controls (Figure 4E), which is in line with increased IL-7R levels following MEF2C induction (Supplemental Figure 4, B and C). Whereas IL-7 signaling is completely inhibited by ruxolitinib, BCL2 levels remain equally high following MEF2C induction and do not change by active IL-7R signaling or following ruxolitinib inhibition (Figure 4E). Therefore, the BCL2 level in LOUCY ETP-ALL cells is directly regulated by MEF2C and does not require active IL-7 signaling.

### MEF2C blocks T cell development.

**Figure 5:**
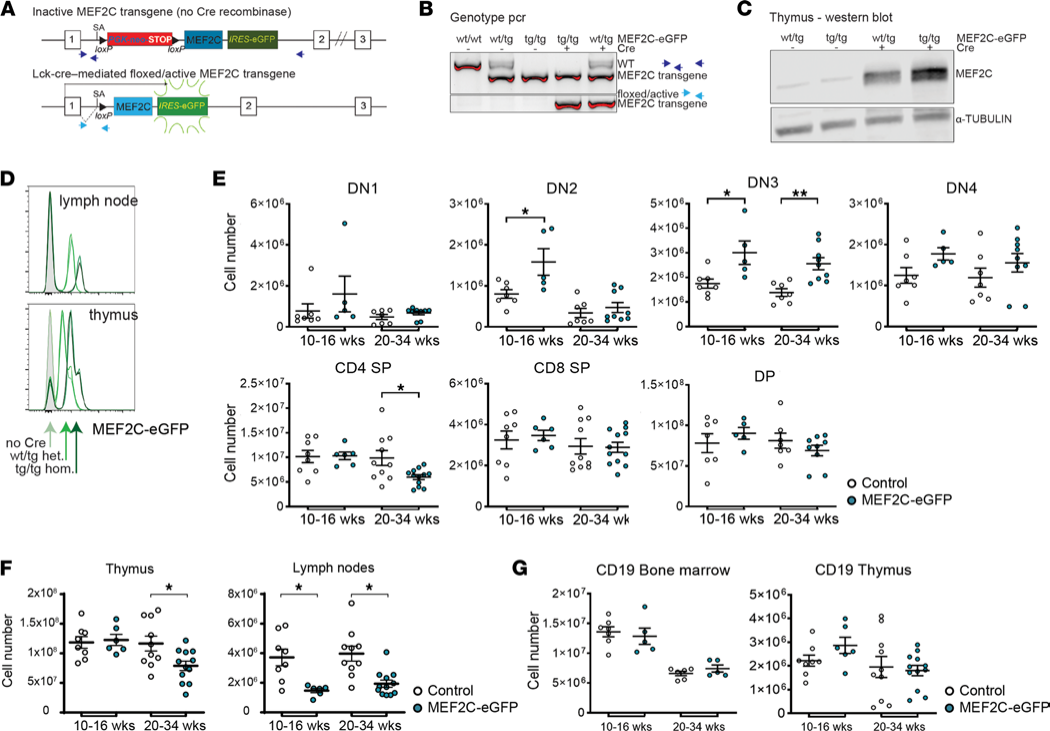
MEF2C blocks T cell development. (**A**) MEF2C-eGFP transgenic mice: loxP-flanked stop cassette followed by MEF2C cDNA, IRES, and eGFP following exon 1 of the Rosa26 locus (top). Cre recombinase activity removes the stop cassette and activates expression of MEF2C and eGFP from the Rosa26 promoter (bottom). SA, splice acceptor site; arrows, genotyping primers. (**B**) Genotype of spleens of mice with WT or MEF2C-eGFP transgene (tg) alleles, in the absence or the presence of Lck-Cre. (**C**) Western blot of endogenous or induced MEF2C and α-tubulin protein levels in total thymocytes from mice of different genotypes. (**D**) Flow cytometry histograms of eGFP expression in lymph node or thymus cells from duplicate MEF2C-eGFP hetero- or homozygous mice with or without Lck-Cre as indicated by the gray, light green, and dark green histograms. (**E**) Absolute cell numbers (mean ± SD) for cells from MEF2C-eGFP control (no Cre, open circles) and MEF2C-eGFP/Lck-Cre mice (blue filled circles) at 10–16 and 20–34 weeks of age. Populations are CD4/CD8 DN, DP, CD4 SP, and CD8 SP. DN are also gated on lineage^–^ (CD3, B220, CD11b, Ly6G/C, Ter-119) cells to further divide the immature thymocyte populations into DN1-DN4. DN1, CD44^+^CD25^–^; DN2, CD44^+^CD25^+^; DN3, CD44^–^CD25^+^; DN4, CD44^–^CD25^–^. (**F**) Total CD45^+^ cell numbers (mean ± SD) as in **E** in the thymus (left panel) or inguinal lymph nodes (right panel). (**G**) Total CD19^+^ cell numbers (mean ± SD) as in **E** in the BM (left panel) or thymus (right panel). *P < 0.05, **P < 0.01 by Mann-Whitney *U* test.

We then studied the effects of ectopic MEF2C expression on T cell development in vivo. For this, we generated *MEF2C-eGFP* conditional knockin transgenic mice in the *ROSA26* locus that were bred on the T cell–specific *Lck-Cre* recombinase transgenic background (Figure 5, A and B). In these mice, MEF2C is induced in early thymocytes and T cells (Figure 5, C and D). At various ages, we observed a modest increase in DN2 and DN3 thymocyte populations compared with control mice, while thymic CD4 counts were reduced in older mice (Figure 5E and Supplemental Figure 6). This is indicative of a partial developmental block. Consistent with this, the numbers of mature CD4^+^ and CD8^+^ T cells in the lymph nodes and spleen are reduced by more than half in MEF2C–conditional knockin mice compared with control mice. Whereas total thymocyte numbers seem normal in younger mice below 16 weeks of age, these numbers significantly decline beyond 20 weeks of age (Figure 5F and data not shown). Because MEF2C overexpression was controlled by the *Lck* promotor, total CD19^+^ B cell numbers were not affected (Figure 5G), indicating that immature T cells could not escape into the B cell lineage.

**Figure 6:**
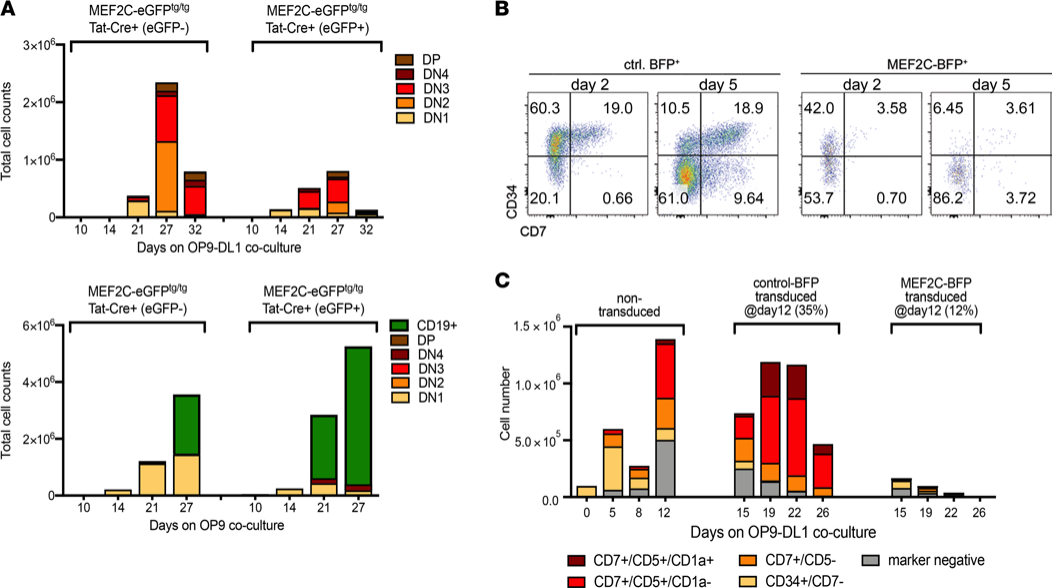
MEF2C blocks T cell lineage differentiation in murine and human cocultures. (**A**) Mouse differentiation assay: lineage-depleted (CD3, B220, CD11b, Ly6G/C, 7-4, Ter-119) BM cells from MEF2C-eGFP^tg/tg^ mice were exposed to Tat-Cre before a coculture experiment (day 0) on OP9-DL1 or OP9 stromal cells to monitor T and B cell differentiation, respectively. (**B**) Human differentiation assay: human CD34^+^ cells from healthy umbilical cord blood donors transduced with BFP control (left) or MEF2C-t2a-BFP (right) lentiviruses and cocultured on OP9-DL1 stromal cells for 2 or 5 days. (**C**) Transduction as in **B**, but at day 12 after splitting the coculture. CD45^+^ cell numbers of a representative example of 3 independent coculture experiments are displayed. For transduced cells, the BFP^+^ percentage is indicated.

We then validated the potential of MEF2C to induce T cell differentiation arrest at early thymocyte stages ex vivo. For this, we isolated BM lineage^–^ progenitor cells from MEF2C^tg/tg^ mice that were incubated with recombinant Tat-Cre protein to express MEF2C-eGFP prior to coculturing experiments on OP9-DL1 stroma. MEF2C-expressing progenitors are less efficient in differentiating toward the T cell lineage compared with control progenitor cells but more efficiently differentiate toward CD19^+^ B cells on OP9 stroma (Figure 6A). Also, human hematopoietic progenitor cells isolated from umbilical cord blood and transduced with the *MEF2C* lentiviral expression construct do not differentiate into the T cell lineage when cultured on OP9-DL1 stroma. In contrast, nontransduced and mock-transduced controls readily give rise to CD7^+^ early T cell subsets (Figure 6B). To further explore whether MEF2C can block T cell differentiation in established early thymocyte stages, we then transduced early CD7^+^CD5^+^ T cells from day 12 OP9-DL1 cocultures; whereas control-transduced cells continue to differentiate toward further T cell stages, MEF2C-transduced cells are completely blocked and disappear over time (Figure 6C). Therefore, expression of MEF2C is incompatible with T cell lineage differentiation.

### MEF2C induces tumors in mice.

**Figure 7:**
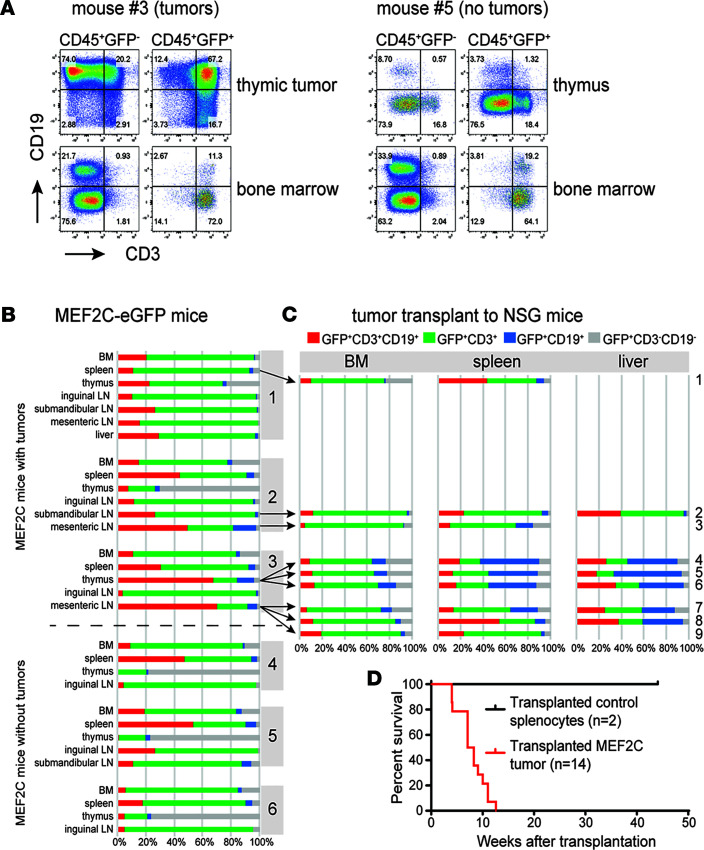
MEF2C induces tumors in mice. (**A**) Flow cytometry analysis of CD3^+^ and/or CD19^+^ cell fractions of CD45^+^ thymus or BM cells from *MEF2C-eGFP*/*Lck*-Cre mouse #3 (with tumor) and mouse #5 (no tumor). (**B**) Summary of CD3^+^ and/or CD19^+^ cell fractions of GFP^+^CD45^+^ cells from 3 *MEF2C-eGFP*/*Lck*-Cre mice with tumors (mice #1–#3) and 3 mice without evidence of tumor growth (mice #4–#6). Percentages of different cell populations are indicated: CD3^+^CD19^+^ (red), CD3^+^ (green), CD19^+^ (blue), and CD3^–^CD19^–^ (gray) cells. (**C**) Summary of CD3^+^ and/or CD19^+^ cell fractions of GFP^+^CD45^+^ cells as in **B** in NSG mice that were transplanted with tumors from indicated tissues of MEF2C-eGFP mice #1–#3 (arrows). Nine of 14 transplanted NSG mice are shown. (**D**) Survival curves of 14 NSG mice transplanted with *MEF2C-eGFP*/*Lck*-Cre tumors (red line) and 2 control NSG mice transplanted with splenocytes from 2-year-old MEF2C-eGFP/no-Cre control mice (black line).

Aging MEF2C/*Lck*-Cre mice developed lymphoid tumors in various organs including the thymus and lymph node areas in 3 of 9 mice at 2 years of age (Figure 7, A and B, and Supplemental Table 3). These tumors are MEF2C^+^ (GFP^+^) and express CD19 (with and without coexpression of CD3) even though MEF2C expression has been induced by the T cell–specific *Lck-*Cre transgene (Figure 7, A and B). Sanger sequencing of the proline (P), glutamic acid (E), serine (S), and threonine (T) (PEST) domain of Notch1 revealed that none of the tumors carried *Notch1*-activating mutations (data not shown). While all tumors express MEF2C, infiltrating GFP^–^CD19^+^ B cells are also found (Figure 7A). BM morphologies for all mice seemed normal, but low percentages of CD3^+^CD19^+^GFP^+^ populations were identified, potentially indicating preleukemic cells even in mice without overt leukemia (Figure 7A). Transplantation of murine MEF2C tumors into immunocompromised NOD-scid IL2Rg^null^ (NSG) mice resulted in rapid infiltration in BM, spleen, and liver (Figure 7C), leading to death within 12 weeks after transplantation (Figure 7D). Some CD3^+^CD19^+^ tumors lost CD3 expression upon transplantation and may undergo similar MEF2C-controlled mechanisms that impair surface CD3/TCRγδ receptor expression, as we observed in LOUCY cells (Figure 7C). The biphenotypic marker expression of these murine tumors is a hallmark of patients with ETP-ALL, as ETP-ALL blasts express early myeloid markers along with T cell markers (1, 23). B cell markers including CD19 have sporadically been reported for patients with ETP-ALL (55, 56). Upon reexamination of the immunophenotype of our historic pediatric ETP-ALL cases, we identified 2 of 13 patients with ETP-ALL for whom immunophenotypic data were available and who expressed CD19 in contrast to 0 of 74 patients with T-ALL (*P* = 0.021, Fisher’s exact test, Supplemental Table 4). MEF2C-eGFP mice, therefore, recapitulate patients with ETP-ALL with multilineage marker expression similar to human mixed phenotype acute leukemia (MPAL) (57). Overall, patients with ETP-ALL highly expressed MEF2C, which suppresses a Notch-mediated T cell differentiation program but elongates a B cell transcriptional and survival program that transforms ETP cells in the thymus context.

### SIK inhibition blocks MEF2C function.

**Figure 8:**
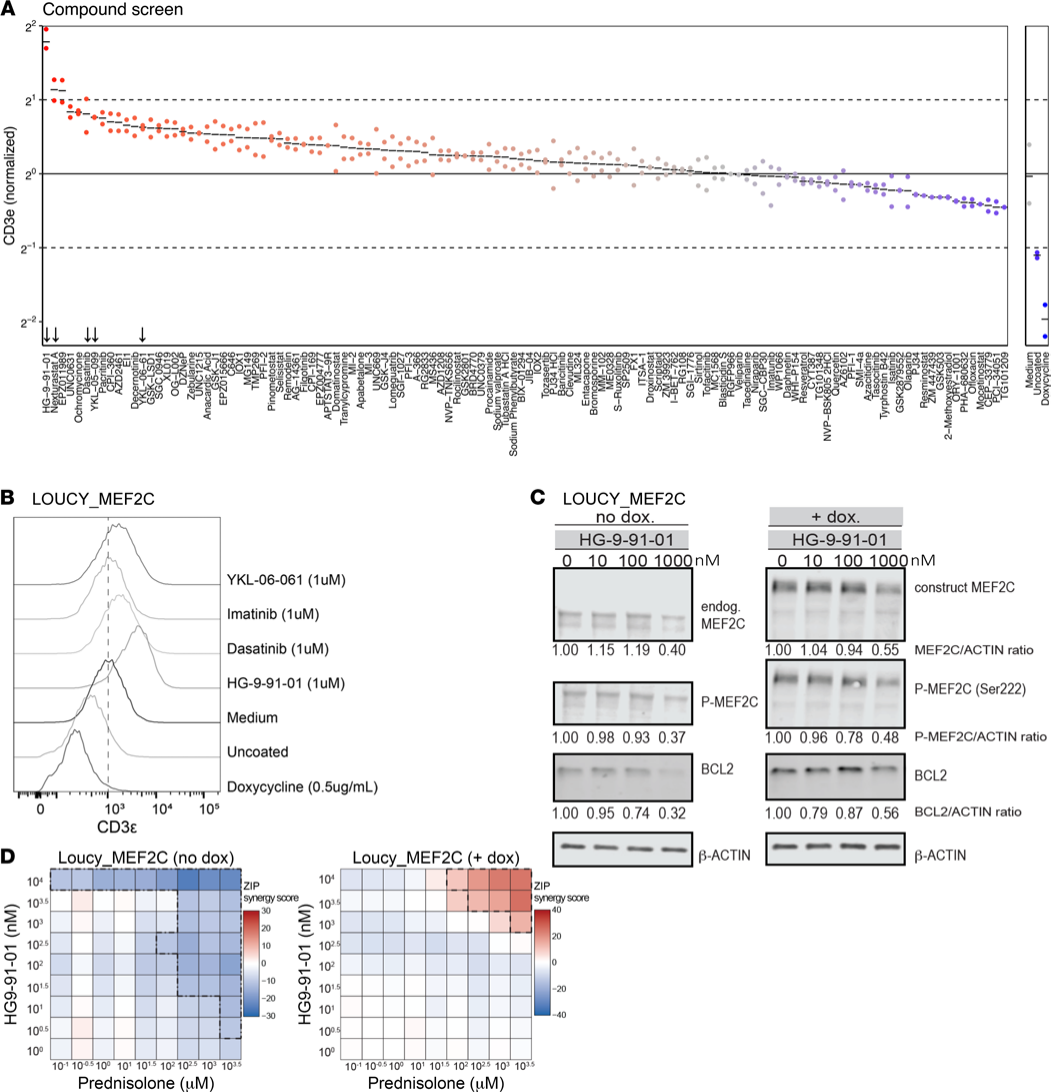
SIK inhibitors block MEF2C function. (**A**) Effects of the compounds from the Selleck Epigenetic Compound Library and various additional inhibitors (1 μM) were screened for their potential to induce a CD3ε^+^ phenotype in LOUCY cells on DLL4-coated plates. As controls, media, non-DLL4–stimulated cells or MEF2C-overexpressing (LOUCY_MEF2C-BFP) cells (+dox) have been indicated in blue. Normalized CD3ε fluorescence intensities have been plotted for duplicate experiments. (**B**) SIK kinase inhibitors YKL-06-062 and HG-9-91-01; dasatinib, which has SIK off-target activity (12 nM, 48 nM, and 180 nM for SIK1, SIK2, or SIK3, respectively) (MedChemExpress or Proteomicsdb.org), and imatinib were tested to induce a CD3ε^+^ phenotype in LOUCY_MEF2C-BFP cells when cultured on DLL4-coated plates in the absence of dox (except when indicated). (**C**) Western blot of MEF2C, P-MEF2C (Ser222), and BCL2 in noninduced (no dox) or MEF2C-induced (+dox) LOUCY_MEF2C-BFP cells that were incubated in the presence of increasing concentrations of inhibitors HG-9-91-01 for 4 days. Band intensity ratios for various proteins relatively to β-actin control levels is indicated, with the –dox condition set at the value = 1. (**D**) Cell viability of noninduced (no dox) or MEF2C-induced (+dox) LOUCY_MEF2C-BFP after a 4-day exposure to a serial dilution of prednisolone combined with a serial dilution of HG-9-91-01 at the concentrations indicated. ZIP synergy scores are calculated from an *n* = 3 per condition. ZIP scores < –10 are indicated with a dash-dot line and a blue color. ZIP scores > 10 are indicated by a dashed line.

MEF2C potently blocks TCRγδ/CD3ε surface expression on LOUCY cells when cultured on OP9-DL1 stroma cells (Figure 2B) or Notch ligand DLL4–coated plates (Figure 8A). Exploiting this feature, we performed a compound library screen using LOUCY cells to identify compounds that can inhibit endogenous MEF2C activity and that drive a TCRγδ^+^/CD3ε^+^ phenotype. As MEF2C is negatively regulated by epigenetic modifiers HDAC4/5 (28), we started screening the Selleck epigenetic drug library plus additional inhibitors for epigenetic regulators (Figure 8A and Supplemental Figure 7). The SIK inhibitor HG-9-91-01 and the HDAC6 inhibitor nexturastat A most potently induced CD3 expression in LOUCY cells. Two other SIK inhibitors (YKL-05-099 and YKL-06-61) were in the top 15 of the strongest MEF2C inhibitors. These results are in line with the normal function of SIK kinases that phosphorylate HDAC4 and HDAC5, thereby alleviating the repression of MEF2C by HDACs (39, 40). Further testing these inhibitors in LOUCY-MEF2C cells cultured in the absence or presence of OP9-DL1 stromal cells validated their potential to enforce surface CD3 expression, even under conditions when MEF2C was highly induced (dox), which normally blocks CD3 expression (Supplemental Figure 8, A and B, and Figure 8B). Dasatinib, which can block SIK kinases at 180 nM (IC_50_) as an off-target effect, also induces a TCRγδ^+^/CD3ε^+^ phenotype in LOUCY cells, whereas imatinib, which lacks such an off-target effect, does not (Supplemental Figure 8B). SIK inhibitors are also cytotoxic for MEF2C^+^ AML line MV4-11 cells (Supplemental Figure 8C) in line with results by others (39, 40, 48). Various other T-ALL lines seem sensitive to SIK inhibitor treatment, including KARPAS-45, ALL-SIL, and HSB-2 (Supplemental Figure 8C). To investigate which SIK is expressed in patients with ETP-ALL versus other patients with T-ALL, we predicted SIK activity scores for ETP-ALL and other patients with T-ALL from the St. Jude RNA-Seq data set (6) using the T-ALL–specific interactome based on the SJARACHNe algorithm (58). This interactome analysis predicted high MEF2C and BCL2 activities in patients with ETP-ALL compared with other patients with T-ALL (Supplemental Figure 9, A–C) in line with our earlier results (Figure 1A). The SIK1 activity, but not SIK2/3 activity, is predicted higher in patients with ETP-ALL compared with other patients with T-ALL who have relatively higher predicted SIK2 and SIK3 kinase activities (Supplemental Figure 9, D–F). The SIK inhibitor HG-9-91-01 does not lower phospho-HDAC4/5 levels or restore their nuclear translocation in LOUCY or MEF2C-induced LOUCY cells (Supplemental Figure 8, D and E). At increasing HG-9-91-01 concentrations, MEF2C and phospho-MEF2C protein levels are reduced (Supplemental Figure 8D), even in LOUCY_MEF2C cells following induction of MEF2C expression (Supplemental Figure 8E and Figure 8C). This reduction in MEF2C protein levels is, in part, regulated through inhibition of *MEF2C* transcription (Supplemental Figure 8F). Inhibition of MEF2C by HG-9-91-01 results in decreased expression of MEF2C target genes, including *IL7R* and *BCL2*, and diminished BCL2 protein levels (Supplemental Figure 8F and Figure 8C). HG-9-91-01 treatment increases *CD5* expression, in line with its potential to restore T cell differentiation following MEF2C inhibition (Supplemental Figure 8F).

Inhibition of MEF2C signalling through SIK inhibition resensitizes MEF2C-expressing LOUCY cells to prednisolone treatment (Figure 8D). Similarly, the BCL2 inhibitor venetoclax synergizes with prednisolone treatment in LOUCY_MEF2C-BFP cells (Supplemental Figure 9G). Overall, SIK inhibitors block MEF2C activity that leads to restoration of T cell differentiation potential, reduced expression of MEF2C target genes including BCL2 and sensitization toward steroid treatment.

## Discussion

Thymus seeding progenitors express MEF2C and are considered the earliest population in the thymus (59). In ETP-ALL, MEF2C can be ectopically expressed by various chromosomal aberrations (5). In *KTM2A/MLL*-rearranged AML, MEF2C plays a pivotal oncogenic role (60, 61) boosting self-renewal, tissue invasion, therapy resistance, and relapse (48, 62, 63). Remarkably, most *KTM2A*-rearranged T-ALLs cluster along with other ETP/immature T-ALL cases that are generally characterized by high expression of *MEF2C*, in addition to other stem cell factors, including *HHEX*, *LMO2*, *LYL1*, and *BCL2* (5). Therefore, MEF2C may represent an attractive therapeutic target for both MEF2C-dependent ETP-ALL and AML.

In our LOUCY model for the role of MEF2C in the ETP-ALL context, the modulation (i.e., overexpression) is needed to demonstrate the role for MEF2C in the survival of LOUCY cells under suboptimal growth conditions, including serum deprivation and steroid-induced death. Knockdown of endogenous MEF2C levels further triggers T cell differentiation, but overexpression of MEF2C levels completely abolishes spontaneous differentiation to a CD3^+^/TCRγδ^+^ phenotype. Since MEF2C seems commonly highly expressed in patients with ETP-ALL due to MEF2C or MEF2C-inducing rearrangements (5), MEF2C is also expressed in the subset of patients with ETP-ALL that harbor HOXA-activating rearrangements as a potential collaborative event. LOUCY harbors the HOXA-activating SET-NUP214 chromosomal rearrangement, and in support of their collaborative effects, we previously demonstrated that knockdown of SET-NUP214 in this line induced a similar T cell differentiation (4) as knockdown of MEF2C levels.

MEF2C blocks thymocyte development by repressing a Notch-induced T lineage differentiation program while propagating a B cell transcriptional program. NOTCH1 is a proven and major regulator of T lineage development that blocks B cell development. Therefore, MEF2C and NOTCH1 may represent opposing regulators of B and T lineage decisions that propagate and repress opposite cell fates. These contrary effects between MEF2C and Notch signaling may reflect a more general mechanism that also applies to other tissues, including fibroblast to cardiomyocyte reprogramming (64) and skeletal muscle development (49, 65). In contrast to those latter studies, we found no evidence for direct interactions between MEF2C and NOTCH1 or the Notch-cofactor MAML1. MEF2C or NOTCH1 may repress each other’s function by regulating repressors. In LOUCY, MEF2C induces *BCL6*, a repressor of Notch target genes that determines the left-right asymmetry cell fates during *Xenopus* development (66). In human follicular lymphoma, pharmacological inhibition of BCL6 liberates NOTCH2 expression, resulting in apoptosis (67). In embryonal rhabdomyosarcoma, Notch signaling promotes a self-renewal program in tumor propagating cells by inducing *SNAIL1*, which strongly represses MEF2C (62). Equal opposing effects between NOTCH1 and GATA3 have been described during T cell commitment (68). Since MEF2C controls GATA3 levels in LOUCY cells, MEF2C may block Notch-induced T cell differentiation via GATA3. These opposing functions between MEF2C and Notch signaling may further explain the reduced incidence of Notch-activating mutations in ETP-ALL (9).

By means of a T-lineage–specific conditional knockin MEF2C^tg/tg^/*Lck*-Cre mouse model, we provide evidence that prolonged MEF2C expression is pathogenic during early T cell development in the thymus that leads to biphenotypic lymphoid tumors. Therefore, MEF2C can act as a bona fide oncogene in ETP cells. These tumors coexpress CD3 and CD19, further displaying the multipotent plasticity of these cells to differentiate into non–T cell fates such as B, myeloid, and NK cells (69, 70). This mouse model mirrors patients with ETP-ALL who can express both T and B markers, also referred to as T/B MPAL (55, 56). MPAL is defined by coexpression of 2 or 3 myeloid, T, and/or B cell lineage markers and likely arises from uncommitted hematopoietic progenitors (57, 71). T/B MPAL is particularly rare but originates from uncommitted ETP cells and could be considered as the earliest subset of ETP-ALL. Its genomic landscape resembles that of ETP-ALL, including recurrent *PHF6* and *IL7R* signaling mutations, along with an absence of NOTCH-activating mutations and genetic alterations that are typically found in pre-B-ALL (55, 56).

For oncogenesis, our study pointed to 2 important molecules, the IL-7R and BCL2, that are both regulated by MEF2C. IL-7 signaling is essential for normal lymphoid development (72–74) and is regulated during the ETP-stage by MEF2C, a stage at which cells are not yet receiving Notch signals (75, 76). Recurrent activating mutations in the IL-7R signaling pathway components are more often found in patients with ETP-ALL than other patients with T-ALL (9, 16, 50) and drive steroid resistance (77, 78). High IL-7R signaling creates an oncogenic environment, and transplanted murine thymocytes with a mutant *Il7r* are able to induce an ETP-ALL–like leukemia in recipient mice. However, prolonged expression of *IL7Ra* in ETP cells requires additional lesions (79). In this context, antiapoptotic *BCL2* warrants a MEF2C-induced survival route that also provides growth advantages under suboptimal conditions. In relation to a MEF2C-induced differentiation arrest, sustained high IL-7R expression levels may be involved in the arrest, since normal DN2 thymocytes need to reduce IL-7 levels before upregulation of the αβ T cell commitment factor *Bcl11b* (80, 81). Since MEF2C is normally downregulated during the ETP stage, prolonged or enhanced MEF2C expression may cause supraphysiological IL-7R signaling and BCL2 expression that contributes to the arrest, survival, and enhanced growth of ETP cells resulting in leukemia. Expansion of arrested cells in the thymus further impairs the entry of new thymus seeding progenitor cells and prevents normal thymocyte turnover, which is also a proven and highly transforming condition (26, 82).

In MEF2C^+^ AML, SIK inhibitors seem highly effective and represent a rare example of kinase inhibitors that indirectly inactivate this oncogenic MEF2C transcription factor (39, 40, 48). Our results demonstrate that this also applies to MEF2C^+^ ETP-ALL. Dasatinib exerts a similar effect by virtue of its anti-SIK off-target effect. In contrast to previous studies (39, 40, 48), we did not observe restoration of nonphosphorylated nuclear HDAC4 levels. Therefore, the exact mechanism by which SIK inhibitors inhibit the MEF2C protein level and function in ETP-ALL remains unclear (48, 83). The SIK inhibitor HG-9-91-01 (and also dasatinib) synergistically reversed the MEF2C-induced steroid resistance of LOUCY cells. Despite the limitation of our model in which this synergistic effect required overexpression of MEF2C, these results point to the potential of SIK inhibitors in combination with steroid treatment for patients with ETP-ALL, who generally are more resilient toward steroid treatment. These results warrant further testing of SIK inhibitors in ETP-ALL samples in vitro or in xenograft NSG mouse models to determine the optimal synergistic combination with steroids. SIK inhibition also results in diminished BCL2 levels (through inhibition of MEF2C) and in sensitized LOUCY cells toward steroid treatment as effectively as the BH3-mimetic venetoclax (84, 85).

In conclusion, SIK inhibitors and dasatinib are both highly interesting compounds to test in MEF2C^+^ ETP-ALL, in addition to MEF2C^+^ AML (39). For this, we may benefit from the ample clinical experience with dasatinib treatment of ALL patients with ABL-like kinase fusions.

## Methods

### Cell lines and culture conditions

LOUCY (ACC-394) and HEK293T (ACC-305) cells were purchased at the DSMZ. OP9-GFP and OP9-DL1 cell lines were provided by JC Zúñiga-Pflücker (University of Toronto, Toronto, Canada). The identity of these and the cell lines LOUCY (ACC-394) and HEK293T (ACC-305, DSMZ) were confirmed by DNA fingerprinting, and cells were regularly tested negative for mycoplasma contamination. OP9 cocultures were performed according to the original protocol (86). Recombinant human SCF (10 ng/mL, R&D Systems), FLT3L (5 ng/mL, Miltenyi Biotec), and IL-7 (2 ng/mL, Miltenyi Biotec) were added at the initiation and every 2–3 days upon splitting the human cocultures. Murine recombinant cytokines (Miltenyi Biotec) for coculture were: IL-7 (5 ng/mL) and Flt3L (5 ng/mL). Overnight recovery of BM lineage-depleted cells prior to the start of the coculture was done in Stemspan (Stemcell Technologies) supplemented with recombinant murine TPO (100 ng/mL), SCF (100 ng/mL), and Flt3L (50 ng/mL).

### Isolation of human and murine progenitor cells

Frozen umbilical cord blood cells were thawed and washed, and CD34^+^ progenitor cells were isolated using the CD34 MicroBead Kit UltraPure according to the manufacturer’s procedure (Miltenyi Biotec, 130-100-453). The magnetic separation was performed using 2 MACS columns (Miltenyi Biotec), consistently reaching a CD34^+^ purity of approximately 95%.

Lineage^–^ progenitors from mouse BM were isolated using the Lineage Cell Depletion Kit (Miltenyi Biotec, 130-090-858). Tat-Cre recombinase (MilliporeSigma, SCR508) was added to 0.5 × 10^6^ to 1.0 × 10^6^ progenitor cells/mL in PBS at a concentration of 2 μM and incubated for 20 minutes at 37°C to recombine the floxed stop cassette in vitro. Cells were washed and incubated overnight in serum-free X-VIVO 10 (Lonza, BE04-743Q) supplemented with recombinant human SCF (50 ng/mL, Miltenyi Biotec), TPO (20 ng/mL, R&D Systems), and FLT3L (50 ng/mL, Miltenyi Biotec).

### Mice

Conditional gain-of-function MEF2C-eGFP transgenic mice were generated by cloning the human *MEF2C* cDNA in the *Rosa26* locus (87, 88). MEF2C-eGFP tg mice were backcrossed for more than 8 generations to the C57BL/6J (Charles River Laboratories, no. 632) background and crossed with *Lck*-Cre mice (Taconic, no. 4197: *B6.Cg-Tg[Lck-cre]1Cwi*). Genotyping primer sequences are listed in Supplemental Table 5.

NSG mice (Charles River Laboratories, no. 614: NOD.Cg-Prkdc^SCID^ Il2rg^tm1Wjl^/SzJ) were used to transplant tumors from MEF2C-eGFP transgenic mice. Six-week-old female NSG mice were injected in the tail vein with MEF2C-eGFP lymph node or thymic tumor cells ranging from 5 × 10^4^ to 8 × 10^6^ per recipient.

Mice were housed in individually ventilated cages in the animal facilities of the Erasmus MC Rotterdam and the Hubrecht Institute Utrecht under specific pathogen–free conditions and in accordance with animal welfare, FELASA (Federation of European Laboratory Animal Science Associations), ethical, and institutional guidelines. Sentinel mice were housed in the same room and tested regularly for a range of bacterial, fungal, viral, and parasitic infections. All animal work was performed under animal experiment permits 103-09-07, 103-12-01 (Erasmus MC), and AVD3990020173065 (Hubrecht Institute).

### Constructs and lentiviral transduction

ETP-ALL LOUCY cells were genetically modified using lentiviral transduction and subsequent flow sorting. Gateway-compatible assembly of lentiviral vectors, virus production, and transduction was performed according to our protocol (89). MEF2C-BFP dox-inducible cells contain the construct: TRE3G_MEF2C_3xHA-T2A-BFP-WPRE-SFFV_TETon-T2A-PuroR in which the human MEF2C was codon optimized and lacks the β TAD domain. MEF2C IPTG-inducible knockdown cells contain a modified version of pLKO-puro-IPTG-3xLacO (MilliporeSigma) in which the puromycin resistance cassette was replaced with GFP. *MEF2C* shRNA was cloned in and resulted in the final construct: U6 RNA promoter-Lac operon-shRNA *MEF2C 3′UTR*-hPGK-GFP-F2A-LacI-WPRE. The sequences of the complementary MEF2C shRNA oligos with *Acc65I* and *EcoRI* overhangs used were: 5′-TACCGGGCCTCAGTGATACAGTATAAACTCGAGTTTATACTGTA TCACTGAGGCTTTTTG-3′ and 5′-AATTCAAAAAGCCTCAGTGATACAGTATAAACTCGAGTT TATACTGTATCACTGAGGCCCG-3′. Identity of the hairpin was confirmed using Sanger sequencing. Dox and IPTG were used in cell culture at final concentrations of 0.5 μg/mL and 1 mM, respectively.

### ChIP-Seq

LOUCY cells were cultured with or without OP9-DL1 for 1 day before harvesting 5 × 10^6^ cells for each ChIP procedure. ChIP was performed according to manufacturer’s instructions (Simple ChIP Enzymatic Chromatin IP Kit, Cell Signaling Technology, no. 9003), with five 30 seconds on/30 seconds off sonication cycles. The following antibodies were used for overnight incubation and from Cell Signaling Technology: MEF2C (catalog 5030), BRD4 (catalog 13440), H3K27ac (catalog 8173), and H3K4me3 (catalog 9751). DNA library preparation was done using the NEBNext Ultra II DNA Library Prep kit (New England Biolabs, E7103), after which it was submitted for sequencing using the Illumina NextSeq500 platform of the Utrecht Sequence facility (USEQ).

### Inhibitors

The following inhibitors and compound library were used at the indicated concentrations and durations: Prednisolone (Sigma-Aldrich, P6004), HG-9-91-01 (MedChemExpress, HY-15776), YKL-05-099 (MedChemExpress, HY-101147), YKL-06-61 (MedChemExpress, HY-120056), venetoclax (MedChemExpress, HY-15531), dasatinib (Sigma-Aldrich, CDS023389), imatinib (Sigma-Aldrich, CDS022105), ruxolitinib (SelleckChem, S1378), DAPT (SelleckChem, S2215), and the Epigenetics Compound Library (SelleckChem).

### Cell viability assay

Cells were cultured in the presence or absence of dox (0.5 μg/mL) for 3 days, washed, and seeded in triplicate at 0.3 × 10^6^ cells/mL in the absence or presence of dox as before. Cells were incubated with prednisolone in a range from 0.1 to 316 μM in combination with either HG-9-91-01 or venetoclax in the range of 1 nM to 10 μM. Each drug was also included as a single range, and DMSO was normalized across the plates. Cell viability was evaluated after 4 days using CellTiter Glo 2.0 (Promega, G9242). For the analysis, viability was normalized to untreated controls and analyzed using the Synergy Finder package in R version 4.0.3 (90, 91). Synergy was defined as a ZIP score > 10, while ZIP scores < –10 were considered antagonistic.

### Compound screen

Ninety-six–well flat-bottom plates were coated overnight at 4°C with 100 μL PBS containing 3 μg/mL rhDLL4 (AcroBiosystems, DL4-H5259). LOUCY_MEF2C-BFP were seeded in duplicate at a concentration of 0.1 × 10^6^ cells/mL and exposed to compounds at a final concentration of 1 μM for 3 days. As controls, cells were left untreated (medium control), treated with dox (0.5 μg/mL) for the MEF2C-induced condition, or seeded in PBS-coated wells (uncoated control). For flow cytometric analyses, geometric mean fluorescence intensities for different compound conditions were determined using FlowJo (v10.7.1) and normalized to the medium control. Data were plotted using R and ggplot2 (92).

### Flow cytometry and antibodies

Flow cytometry was performed using the Cytoflex (Beckman Coulter) or the BioRad ZE5 flow cytometer, and data analysis was performed using the FlowJo software (version 10.6, TreeStar Inc.). Human flow antibodies were obtained from Miltenyi Biotec unless specified otherwise: CD3ε (clone REA613), TCRγδ (clone 11F2), CD5 (clone UCHT2), CD34 (clone AC136), CD45 (clone 5B1), CD127 (IL-7Ra, clone MB15-18C9), CD1a (BD clone HI149), CD7 (BD clone M-T701), and NOTCH1-PE (BioLegend, 352105). Mouse flow antibodies were obtained from BioLegend unless specified otherwise: CD3ε (clone 145-2C11), CD4 (clone GK1.5), CD8 (clone 53-6.7), CD19 (clone 6D5), CD25 (BD clone PC61), CD44 (BD clone IM7), and CD45 (Miltenyi Biotec, clone 30F11).

### Western blot antibodies

Western blot antibodies were obtained from Cell Signaling Technology unless specified otherwise: MEF2C (catalog 5030), Cleaved NOTCH1 Val1744 (catalog 4147), MAML1 (catalog 11959), STAT5 (catalog 94205), phospho-STAT5 Tyr694 (catalog 9351), HA-tag (catalog 3724), DDK-tag (catalog 2368), HDAC4 (catalog 15164), phospho–HDAC4 Ser246/HDAC5 Ser259/HDAC7 Ser155 (catalog 3443), BCL2 (Santa Cruz Biotechnology Inc., sc-130308), LAMIN B (Santa Cruz Biotechnology Inc., sc-6216), β-actin (Abcam, ab6276), phospho-Ser222 MEF2C (PhosphoSolutions, p1208-222), and SIK3 (Sigma-Aldrich, HPA048161).

### Bioinformatic analyses

#### Affymetrix gene expression arrays.

Integrity of total RNA was verified using the Agilent 2100 Bioanalyzer (Agilent Technologies). Copy RNA synthesis, hybridization to HG U133 plus 2.0 microarrays (Affymetrix), and subsequent steps were performed according to the manufacturers’ protocol. Data analysis was performed in R version 3.6.3. The Affy package (93), version 1.64.0, was used for Robust Multichip Average (RMA) normalization. All +dox samples (*n* = 9) were compared with the –dox samples (*n* = 9), using the Limma package (94), version 3.42.2. Upregulated (log_2_ fold change > 0.6, *P* < 0.05, and *q* < 0.1) and downregulated (log_2_ fold change < –0.6, *P* < 0.05, and *q* < 0.1) probe sets were collapsed to genes. The gene names were used for GO term analysis in the web version of PANTHER (released 20200407, ref. 95), using the human data set with default settings. Heatmaps were created using Pheatmap package in R.

### Cluster analysis of the 264 pediatric and young adult T-ALL series

From the 416 genes that yielded robust unsupervised clusters for 117 pediatric T-ALL patient samples (5), 371 genes could be matched in the RNA-Seq data set of 264 patients with T-ALL (6) (Supplemental Table 1). Visualization and heatmap were constructed using “Pheatmap” package in R, version 1.0.12 Raivo Kolde. In order to extrapolate differences and avoid outliers in the colors, breakpoints in colors are defined at black to gray (–1 to 1), dark to light blue (1 to 3), yellow to dark orange (3 to 6), and red to dark red (6 to 13).

#### ChIP-Seq data processing and visualization.

Raw reads were aligned to the HG19 genome using Burrows-Wheeler-Aligner (BWA), with default settings. MACS2 was used for peak calling. Broad peak calling was used for histones, and narrow peak calling was used for transcription factors. The remaining were default settings. Peaks were visualized using IGV viewer (96). Centered heatmaps were created using Deeptools (97). Region 5 kb upstream of transcription start site (uTSS) were taken from HG19 using Bedtools (98). Enhancers (Enh) were downloaded from GeneHancer (99), and the 5 kb upstream regions of long noncoding areas (uLnc), as defined by deepbase (100) and lncipedia (101), were used. Other regions are binding sites that did not fall in uTSS, Enh, or uLnc areas. Genomic locations of up- and downregulated genes, described above, were retrieved from HG19. Binding sites within a window of 10 kb from a up- or downregulated gene were selected using Bedtools. Motifs were detected using MEME-ChIP (102) within 50 bps up or down from the MEF2C peak summit (101 bp window) using default settings (Supplemental Figure 3).

#### Construction of T-ALL interactome via SJARACNe and activity score estimation.

To reverse engineer a T-ALL–specific interactome (TALLi), we applied SJARACNe (58) with default parameters (bootstrap = 100; consensus cluster, *P* = 1 × 10^–5^) to the TARGET T-ALL RNA-Seq data set (6) using the expression profile of 27,218 unique genes from a total of 261 patients. We removed 5820 genes with low or invariable expression across samples and 3 outlier patients. Based on Gene Ontology (GO) classification, we compiled a list of transcription factor genes and signaling molecule genes (*n* = 2002 and 9626, respectively). Transcription factor network and signaling molecule network were generated separately using SJARACNe, with drivers (hubs) linked to their predicted targets through interactions (edges) based on gene-to-gene relationship derived from their expression pattern. After combining these 2 networks, the final data-driven TALLi consisted of 35,102 nodes (genes) and 1,068,228 edges, including 7924 unique hub genes consisting of 1653 transcriptional factors and 6271 signaling molecules. Cal.Activity function (method = “weightedmean”) in NetBID was employed to infer the activities of driver genes for each patient from their gene expression profiles and the TALLi. The weighted mean activity of a hub (driver) gene “i” in sample “s” was defined by the following equation: 
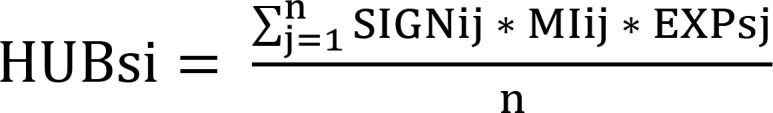
. The gene expression matrix was Z-normalized in each sample, and EXPsj is the expression value of gene “j” in sample “s”. MIij is the mutual information between driver gene “i” and its target gene “j”, and SIGNij is the sign of spearman correlation between gene “i” and its target gene “j”. The total number of targets for driver “i” is denoted by “n”. The MEF2C, BCL2, SIK1, SIK2 and SIK3 scores are based on 178, 145, 118, 105, and 40 genes, respectively (Supplemental Table 6).

### Data and code availability

The 264 pediatric and young adult T-ALL St Jude RNA-Seq data (6) with accession no. PHS000464 were downloaded from the target database (https://ocg.cancer.gov/programs/target). The Affymetrix U133 Plus2 microarray data set for 117 patients with T-ALL is available at http://www.ncbi.nlm.nih.gov/geo/ under accession no. GSE26713 (5). The Microarray U133 Plus2 data set for LOUCY and LOUCY_MEF2C-BFP lines such as used in this study have been deposited in NCBI’s Gene Expression Omnibus under accession no. GSE159506. The ChIP-Seq files are available under accession no. GSE160409.

### Human subjects

Diagnostic patient biopsies of children who have been diagnosed with T-ALL were provided by the German Cooperative study group for Childhood Acute Lymphoblastic Leukemia (COALL) and the Dutch Childhood Oncology Group (DCOG). Immunophenotype data were used from diagnostic T-ALL patient samples, for which Affymetrix U133 plus 2 gene expression microarray data were also available. Positivity levels for CD markers are set at 25% (Supplemental Table 4).

### Statistics

Nonnormally distributed continuous data were analyzed using the Mann-Whitney *U* test. The Fisher’s exact or log-rank test was used to calculate significant differences in the distribution of ETP-ALL or other T-ALL subtype over different immunophenotypic or genetic categories as indicated. Pearson’s correlation and *R*^2^ were determined to calculate correlations between *LMO2* or *BCL2* and *MEF2C* relative expression levels. Mouse population cell numbers and MEF2C-induced gene expression levels in LOUCY or LOUCY_MEF2C-BFP cells were analyzed using the 2-tailed Student’s *t* test.

### Study approval

The patients’ parents or legal guardians provided informed consent to use leftover diagnostic materials for research purposes in accordance with the Declaration of Helsinki. This study was approved by the Institutional Review Board of the Erasmus MC Rotterdam. Mice were housed in accordance with animal welfare, Federation of European Laboratory Animal Science Associations (FELASA), ethical, and institutional guidelines; all animal work was performed under animal experiment permits 103-09-07, 103-12-01 (Erasmus MC), and AVD3990020173065 (Hubrecht Institute). Human umbilical cord blood samples were obtained after informed consent in accordance with the Declaration of Helsinki and in agreement with the institutional ethics committee.

## Author contributions

KCB conceptualized the study, performed research and validation studies, and wrote and reviewed the manuscript; MTM performed research and validation studies, analyzed data, and wrote and reviewed the manuscript; JJY performed research, analyzed data, and reviewed the manuscript; VC performed research and reviewed the manuscript; RH curated data, performed formal analysis, and reviewed the manuscript; WY, JY, MEN, WKS, and JPJ performed research; JJH and SG created the conditional knockin mouse model and reviewed the manuscript; RP advised and reviewed the manuscript; JPPM conceptualized the study, performed research, supervised the study, and wrote and reviewed the manuscript. The order of co–first authors was determined based on initiation and conceptualization of the study.

## Supplementary Material

Supplemental data

Supplemental table 1

Supplemental table 2

Supplemental table 3

Supplemental table 4

Supplemental table 5

Supplemental table 6

## Version 1 05/10/2022

In-Press Preview

## Version 2 07/08/2022

Electronic publication
