# Supplementary material for: MEF2C opposes Notch in lymphoid lineage decision and drives leukemia in the thymus
Source: JCI Insight. 2022 Jul 8;7(13):e150363. doi: 10.1172/jci.insight.150363 (PMC9310523; doi:10.1172/jci.insight.150363)
Supplement: Supplemental data [file jciinsight-7-150363-s171.pdf]

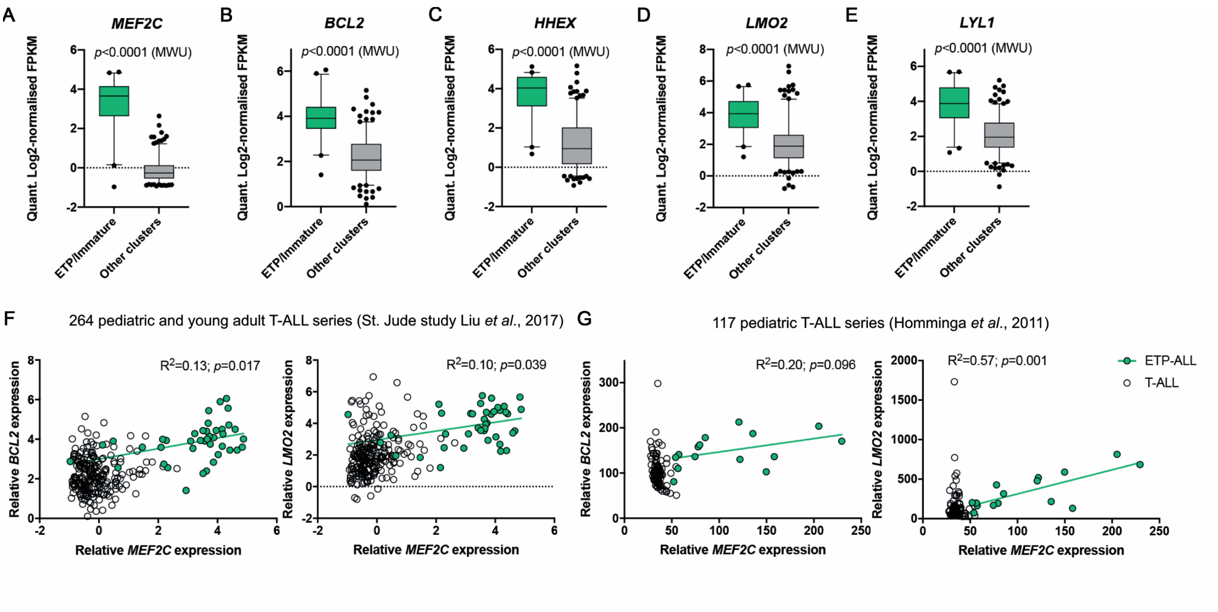

Supplementary Figure S1 (with Figure 1). ETP/immature subtype pediatric and young adult T-ALL patients highly express *MEF2C* and other stem-cell factors *HHEX*, *LMO2*, *BCL2* and *LYL1*. Expression of *MEF2C* (A), *BCL2* (B), *HHEX* (C), *LMO2* (D) and *LYL1* (E) for 41 ETP/immature cluster patients versus 223 other T-ALL patients. The 5- and 95-percentiles have been indicated by whiskers. F. Correlation analyses of relative *BCL2* and *LMO2* expression levels with *MEF2C* expression levels in 41 ETP/immature cluster patients versus 223 other T-ALL subtype patients in the RNA-seq dataset of Liu *et al* (2017) (6). G. Correlation analyses of *LMO2* and *BCL2* with *MEF2C* expression levels in 41 ETP/immature cluster patients versus 223 other T-ALL subtype patients as present in the GEO microarray dataset GSE26713 (5)).

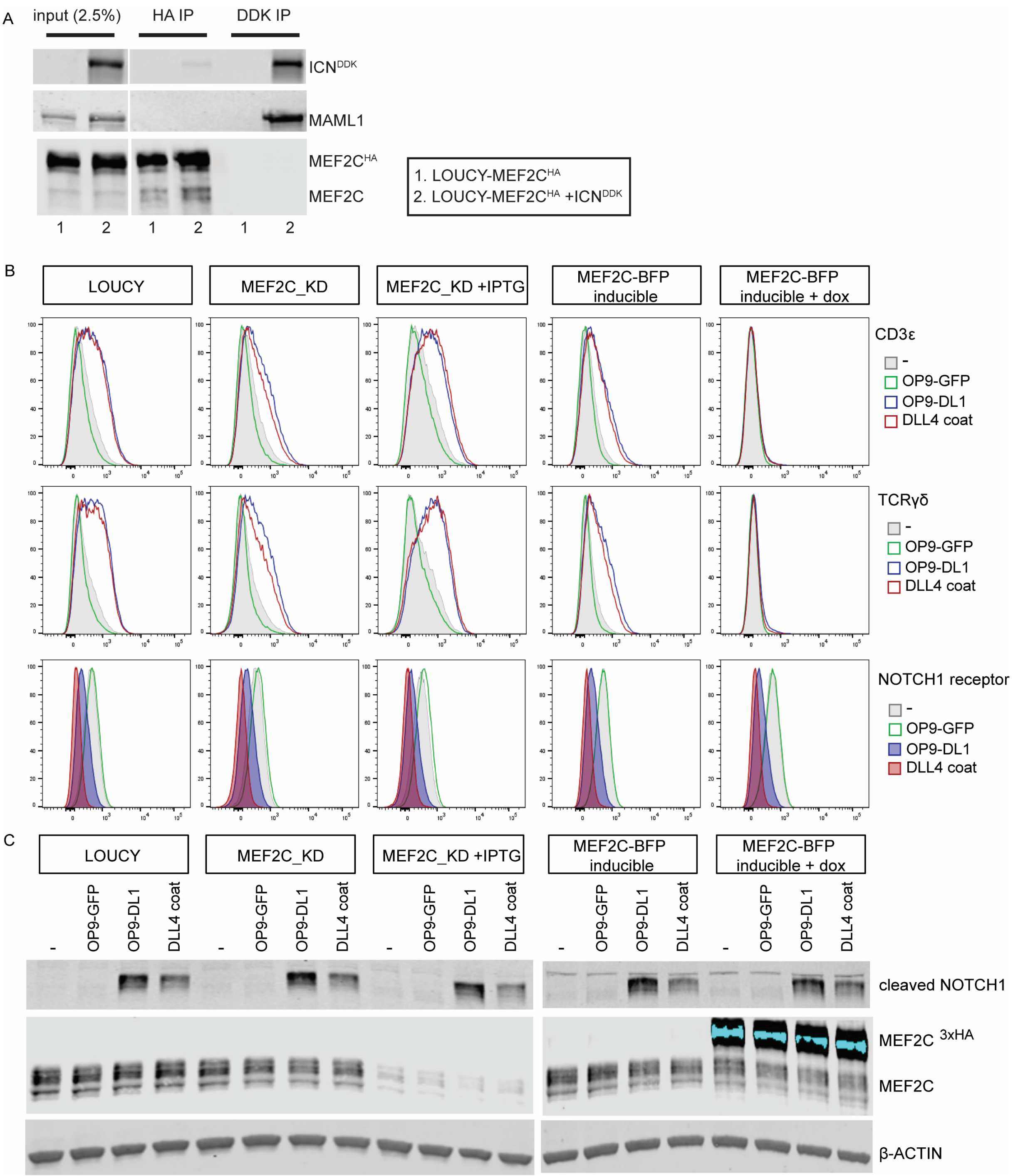

Supplementary Figure S2. **MEF2C and NOTCH1 receptor or intracellular cleaved NOTCH1 (ICN) do neither interact, nor affect each other at the level of protein expression.** A. Western blot of co-immunoprecipitated proteins. MEF2C^{HA} and ICN^{DDK} do not bind directly, nor via MAML1. Two LOUCY lines were used: the MEF2C-BFP doxycycline-inducible line (1.) and this line in combination with a truncated ICN lacking the PEST domain (2.), which contained TRE3G_ICN_3xddk-T2A-VenusGreen-WPRE-SFFV_TETon-T2A-NGFR in addition to the MEF2C inducible vector (see methods). B. Flow cytometry histograms of the LOUCY parent line, MEF2C knock-down (KD) control and +IPTG, and MEF2C-BFP inducible control (-dox) and +dox cell lines. Whereas CD3ε and TCRγδ surface expression is induced by Notch signaling via both OP9-DL1 and plate-coated Delta-like 4 ligand (DLL4 coat), the NOTCH1 receptor surface expression is reduced. C. Western blot of total intracellular cleaved NOTCH1 (ICN) and MEF2C levels in the different conditions and cell lines as in (B). β-actin is used as a loading control. Absence or presence of MEF2C does not affect any of the NOTCH1-related changes in (B) and (C). The results shown are representative examples of at least three independent experiments.

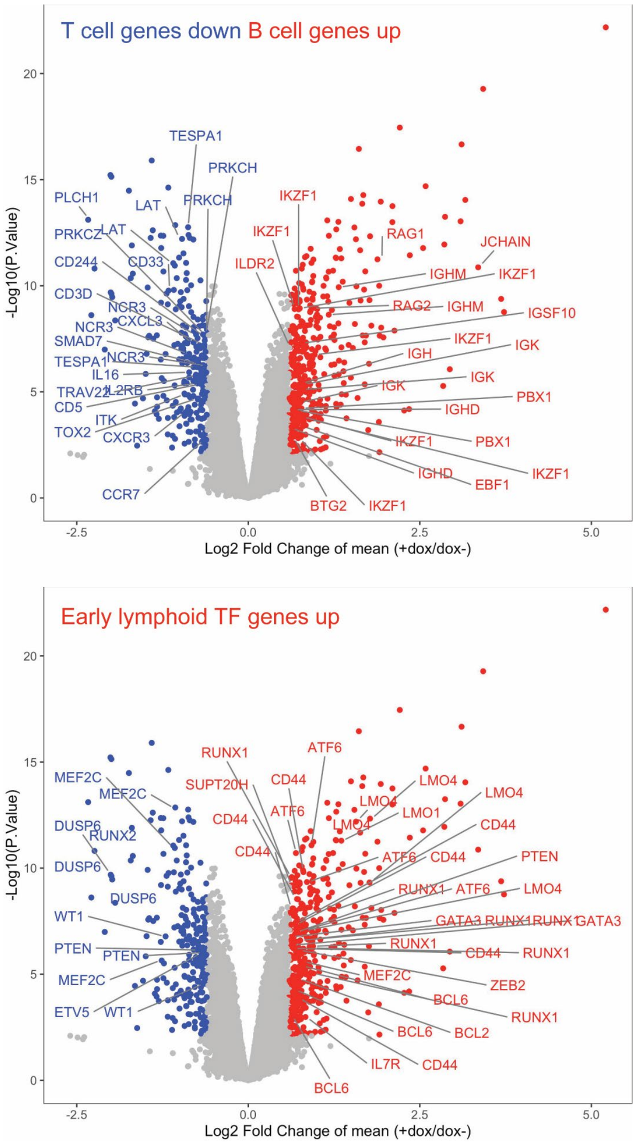

Supplementary Figure S3. **Gene expression in LOUCY cells overexpressing MEF2C.** Related to Figure 1C. Volcano plots with significantly up- or downregulated probesets (log2 fold change >0.6, p<0.05, q<0.1) in LOUCY with/without MEF2C overexpression for 24 hrs (+/- dox.). MEF2C-induced down-regulated (blue) and upregulated (red) probesets, shown for triplicate - and + doxycycline-induced LOUCY_MEF2C samples.

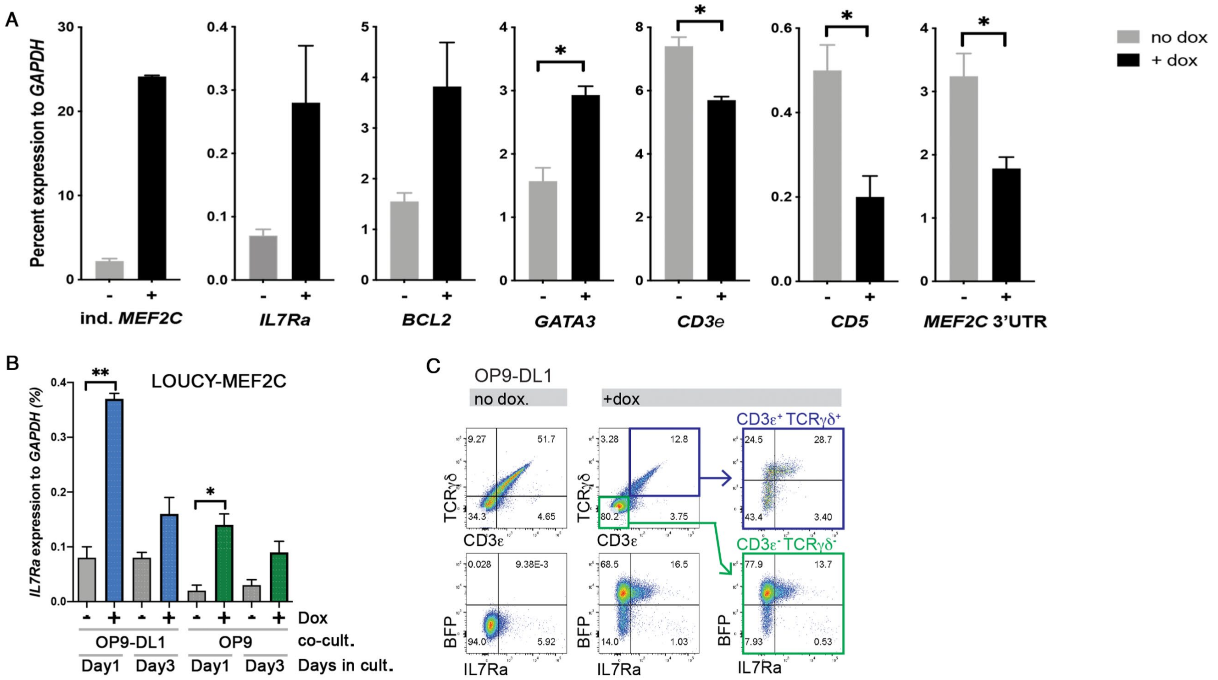

Supplementary Figure S4 (with Figure 2). MEF2C antagonizes Notch signaling in early progenitor T cells and induces B cell gene expression. A. Real-time quantitative PCR (RT-QPCR) validation of select target genes in LOUCY_MEF2C cells that were culture in the absence (grey bars) and presence of doxycycline-induced MEF2C expression (black bars) for 24 hrs. A representative experiment in shown form 3 independent experiments. Mean expression values (from duplicate experiments±SD) for *IL7Ra*, *BCL2*, *GATA3*, *CD3e*, *CD5* and endogenous *MEF2C* (3' UTR) following induction of the MEF2C-BFP construct (Ind.MEF2C) are shown as percentage of *GAPDH* mRNA expression. Significance levels (*p<0.05) by unpaired T-test are indicated. *MEF2C 3'UTR* reflects endogenous *MEF2C* expression levels. Primer sequences are listed in Supplementary Table S5. B. RT-QPCR for normalized *IL7Ra* mRNA levels relative to *GAPDH* levels in LOUCY_MEF2C-BFP cells that are grown under the conditions as indicated. A representative experiment is shown from 3 independent experiments. Significance levels (*p<0.05 or **P<0.01) by unpaired T-test have been indicated. C. Flow cytometry analysis of surface TCRγδ/CD3ε expression, BFP (reflecting induced MEF2C levels) and IL7Ra expression of LOUCY_MEF2C-BFP cells without or with induction of MEF2C (+ dox) that were co-cultured on OP9-DL1 for 1 day. Results for gated TCRγδ$^+$/CD3ε$^+$ (blue gated) and TCRγδ$^-$/CD3ε$^-$ (green gated) cells are shown.

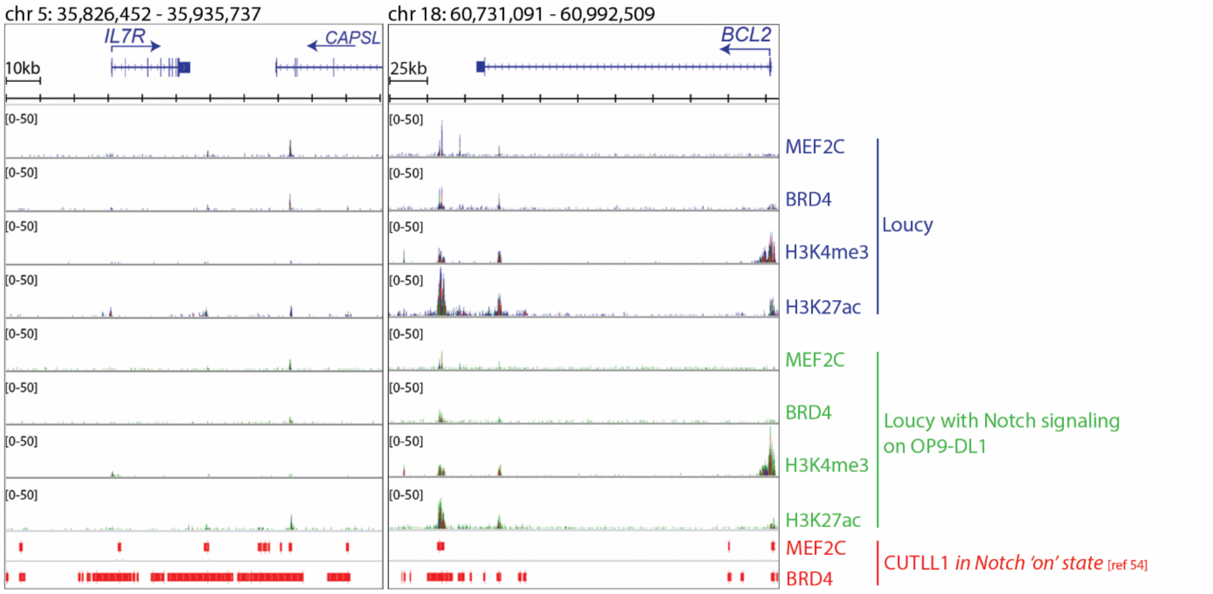

Supplementary Figure S5 (with Figure 3). MEF2C binds to promoters and enhancers, which is reduced by Notch signaling. Chromatin landscape of LOUCY cells as indicated (see A) for the *IL7R* (left) and the *BCL2* (right) genes including binding sites for MEF2C, BRD4 or histone modifications H3K4me3 and H3K27ac. For comparison, the published landscapes with NOTCH1 and BRD4 binding sites in Notch-activated CUTLL1 cell line (54) are shown (GSE51800).

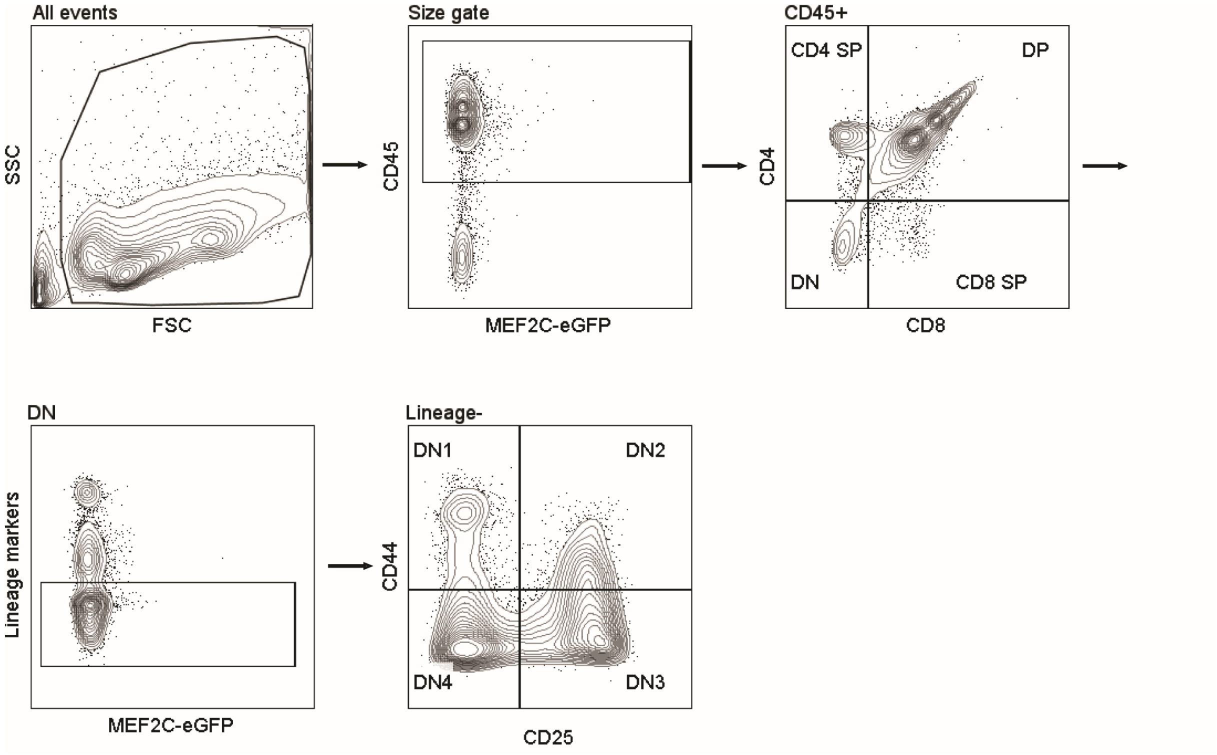

Supplementary Figure S6. FACS gating strategy for mouse immunophenotyping. Upon acquisition, murine cells were gated based on size in an FSC/SSC plot to remove debris. Cells were then gated on CD45. CD45+ cells were categorized as double negative (DN), CD4 single positive (SP), CD8 SP, or double positive (DP) based on CD4 and CD8 expression. The DN population was then gated on lineage-negativity using a dump channel including B220, TER-119, Ly-6G/C and CD11b. CD45+CD4-CD8-Lineage- were then characterized as DN1 (CD44+CD25-), DN2(CD44+CD25+), DN3 (CD44-CD25+) or DN4 (CD44-CD25-).

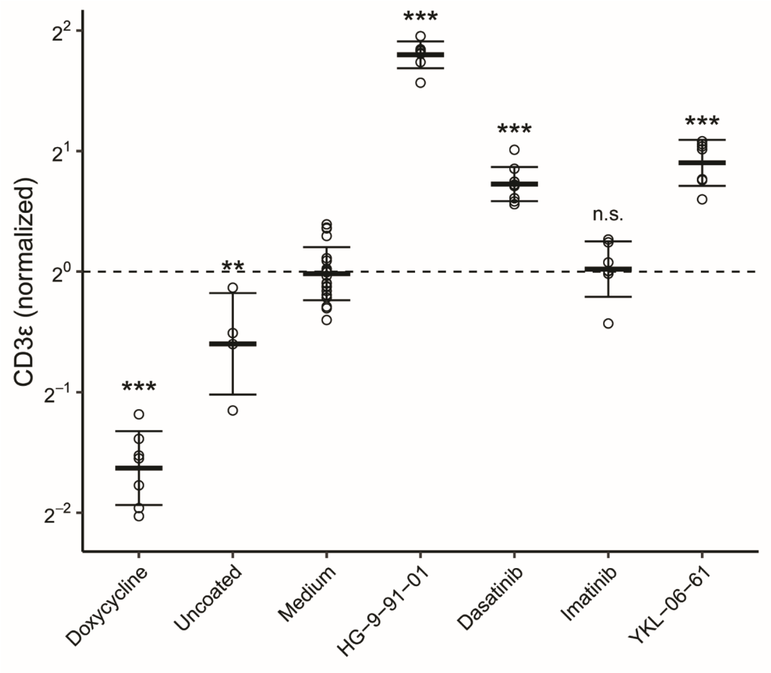

Supplementary Figure S7. In vitro compound screening on LOUCY-MEF2C yields highly reproducible results. Loucy_MEF2C cells were seeded on a plate pre-coated with recombinant human Delta Like Ligand 4 (DLL4) or PBS (Uncoated control). Cells were treated with doxycycline, to induce MEF2C overexpression, or with 1uM on the indicated compounds. After a 3-day incubation, cells were assessed for CD3ε expression using flow cytometry. The geometric mean fluorescent intensity was normalized to the average medium control. Data was pooled from 3 independent experiments. Data is displayed as dot plot, with crossbars indicating the mean and standard deviation. Data was analysed using a one-way ANOVA followed by pairwise t-tests with Holm's correction. *** $p<0.001$,** $p<0.01$, n.s. not-significant; all compared to medium.

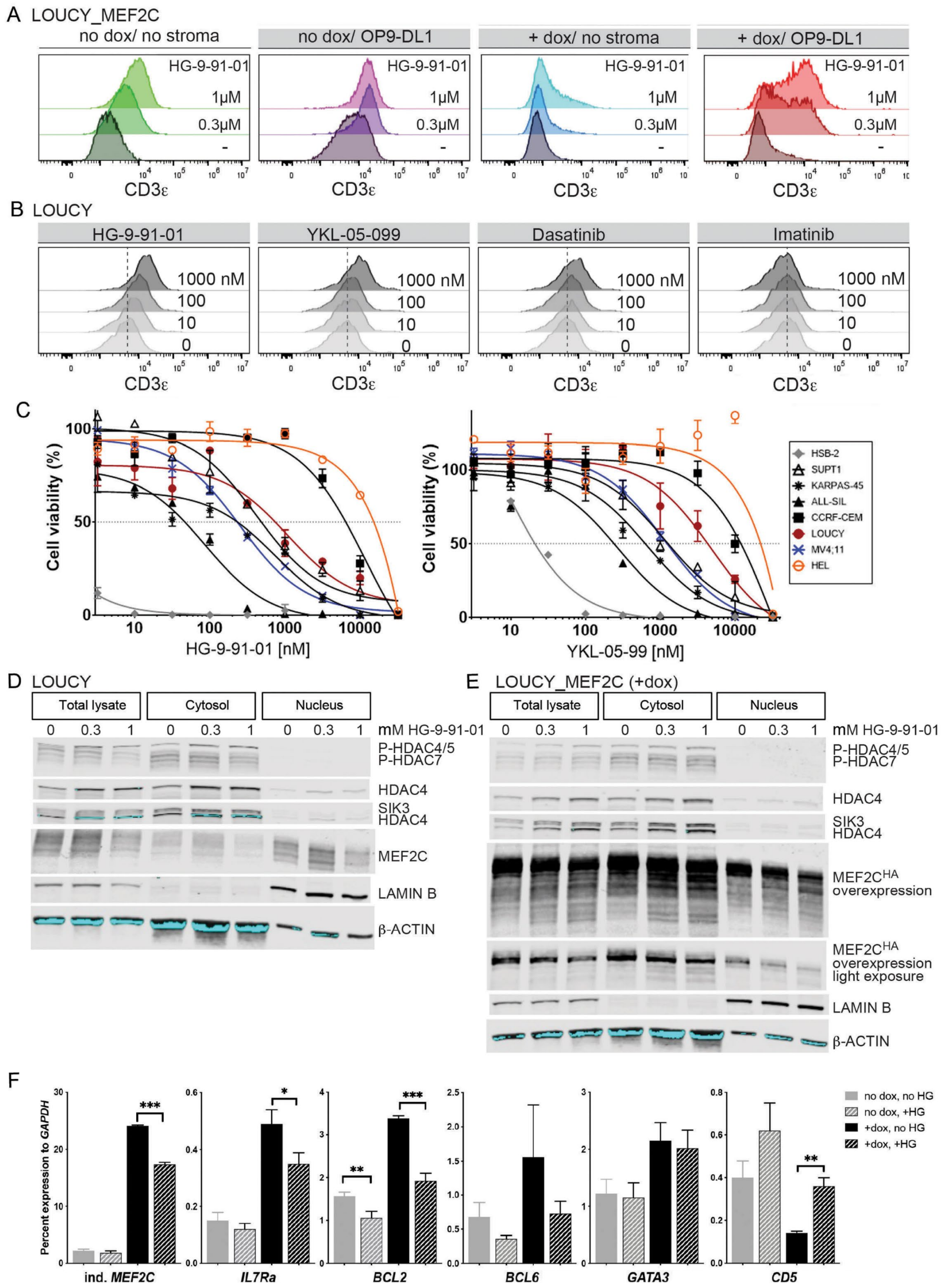

Supplementary Figure S8. **SIK inhibition induces MEF2C-repressed CD3 expression but does not inhibit HDAC phosphorylation or stimulate HDAC translocation to the nucleus.** A. CD3ε surface expression on non-induced (no dox) or MEF2C-induced (+dox) LOUCY_MEF2C-BFP cells that were incubated for 4 days without or with OP9-DL1 stroma support in the absence or presence of 0.3μM or 1μM HG-9-91-01. B. CD3ε surface expression on parental LOUCY cells that were cultured in the presence of increasing concentrations of SIK inhibitors HG-9-91-01 or YKL-05-099, SRC-kinase inhibitors dasatinib or imatinib for 4 days as indicated. C. Cell viability assay for various T-ALL cell lines (CCRF-CEM, LOUCY, SUPT1, KARPAS-45, ALL-SIL and HSB2), the AML cell line HEL and the BCP-ALL cell line MV4;11 that were exposed to a serial dilution of SIK inhibitors HG-9-91-01 (left) or YKL-05-099 (right) for 4-days as indicated. Mean values ±SD are shown for duplicates. D. and E. Western blot analysis of total cell lysates or cell fractions as indicated for LOUCY cells (D) or LOUCY_MEF2C-BFP cells (+doxycycline; E) following a 24hr exposure to SIK inhibitor HG-9-91-01 at concentrations indicated. Blots have been stained for the (phosphorylated) proteins as indicated. F. RT-QPCR normalized expression values (mean ±SD of a triplicate experiment) for various MEF2C target genes relative to *GAPDH* expression values were measured in non-induced (no dox) or MEF2C-induced (+dox) LOUCY_MEF2C-BFP cells that were cultured for 24 hrs in the presence or absence of 1μM HG-9-91-01 as indicated. Ind.MEF2C: induced MEF2C-BFP. Significance levels by unpaired T test have been indicated (*p<0.05, **p<0.01, ***p<0.001, ****p<0.0001). Primer sequences are listed in Supplementary Table S5.

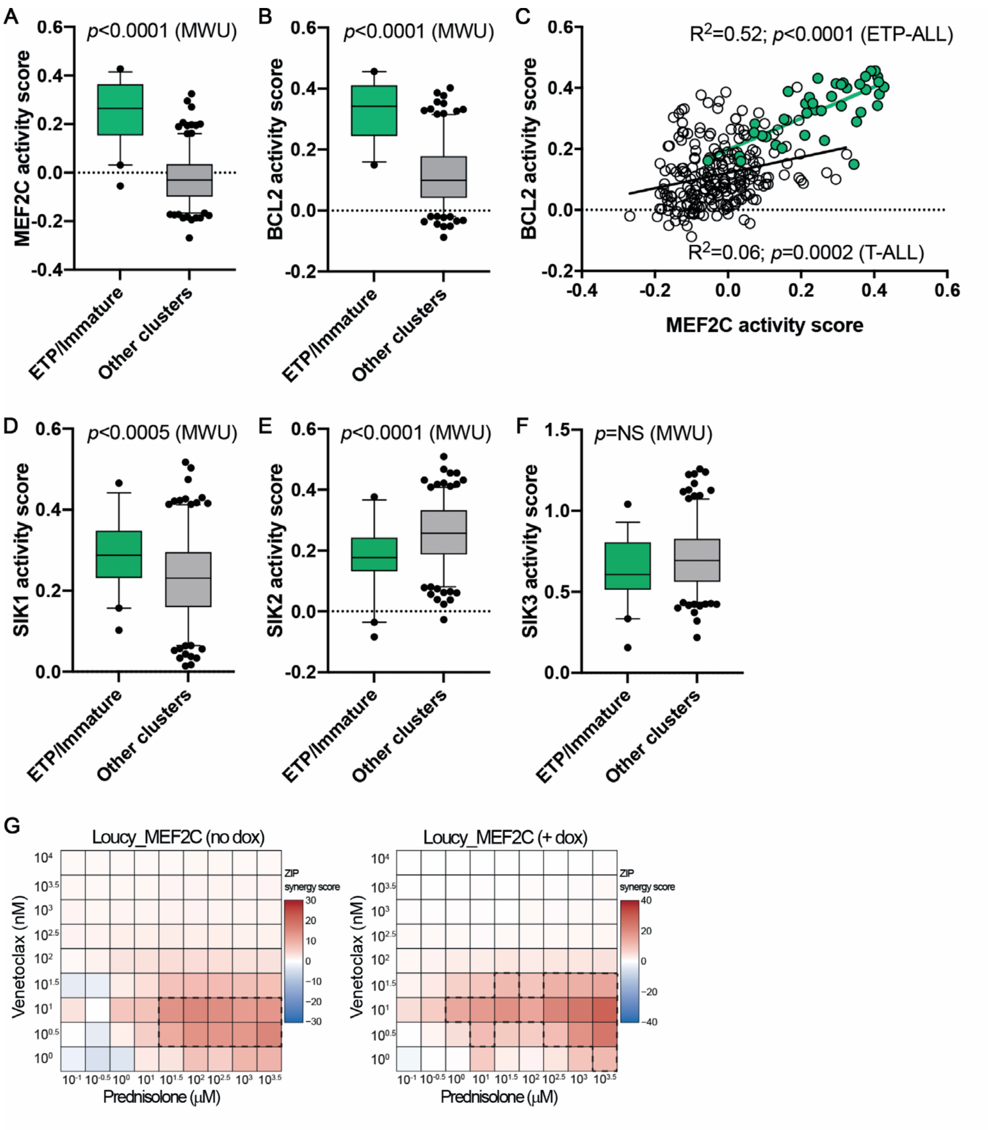

Supplementary Figure S9 (with Figure 7). SIK inhibitors block MEF2C function. A-F. SJARACHNe-predicted activity scores for MEF2C (based on 178 genes), BCL2 (based on 145 genes), SIK1 (based on 118 genes), SIK2 (based on 105 genes) and SIK3 (based on 40 genes) for 39 ETP-ALL patients compared to 221 other T-ALL subtype patients from the RNA seq dataset of 261 pediatric and young adult T-ALL in the St Jude Research Hospital as published by Liu *et al* (2017) (6). The 5- and 95-percentiles in activity scores have been indicated by whiskers. Statistical differences have been calculated using the Mann-Whitney-U test. The correlation between MEF2C and BCL2 activity scores for ETP-ALL versus other T-ALL patients is shown including the R-squared values and the linear regression p-values. G. Cell viability of non-induced (no dox) or MEF2C-induced (+dox) LOUCY_MEF2C-BFP after a 4-days exposure to a serial dilution of prednisolone combined with a serial dilution of venetoclax at the concentrations indicated. ZIP synergy scores are calculated from an N = 3 per condition. ZIP scores <-10 are indicated with a dash-dot line and a blue color. ZIP scores >10 are indicated by a dashed line.
